# Optimized Continuous Thermosonication for Sustainable Pasteurization of Orange and Dried Black Lime Juices: Techno‐Functional, Physicochemical, and Microbial Assessment

**DOI:** 10.1002/fsn3.71171

**Published:** 2025-11-09

**Authors:** Alaa R. Abdulstar, Asaad R. Al‐HiIphy, Ammar B. Altemimi, Rawaa H. Tlay, Tarek Gamal Abedelmaksoud

**Affiliations:** ^1^ Department of Food Science College of Agriculture, University of Basrah Basrah Iraq; ^2^ Department of Food Science, College of Agricultural Engineering Damascus University Damascus Syria; ^3^ Food Science Department, Faculty of Agriculture Cairo University Giza Egypt

**Keywords:** emerging technology, microbiological safety, pectin methylesterase, response surface methodology, RP‐HPLC, sustainability

## Abstract

In pursuit of sustainable and efficient food processing solutions, a batch thermosonication system was successfully upgraded into a continuous thermosonication system (CTS) to improve pasteurization performance for orange juice (OJ) and dried black lime juice (DBLJ). The newly developed CTS integrates five core components: thermal heating, pumping, heat exchange, pasteurization, and electrical control units. Using response surface methodology, the effects of power (1.676–1.764 kW), mass flow rate (0.0142–0.0016 kg/s), and processing temperature (30°C–50°C) on the techno‐functional, physicochemical, and microbiological attributes of the juices were optimized. At optimal conditions, the CTS reduced specific energy consumption by 29.71% in OJ and 30.51% in DBLJ, relative to traditional pasteurization (TP: 90°C for 60 s), while improving energy efficiency and process productivity. No significant differences (*p* > 0.05) were observed in pH, titratable acidity, or sensory characteristics compared to untreated fresh juice. Moreover, the residual activity of pectin methylesterase was effectively reduced to 7.48% ± 0.11% (OJ) and 6.42% ± 0.074% (DBLJ). Microbiological analysis revealed complete inactivation of total bacterial count, psychrotrophic bacteria, coliforms, and yeasts and molds. Additionally, RP‐HPLC analysis confirmed enhancement in the bioactive compound content in CTS‐treated juices compared to both fresh and TP samples. These findings demonstrate that the CTS approach offers a sustainable, energy‐efficient, and quality‐preserving alternative for juice pasteurization, highlighting its potential application in modern food processing industries.

## Graphical abstract

The study developed a continuous thermosonication system (CTS) for the sustainable pasteurization of orange and dried black lime juices. CTS reduced energy consumption by 30% compared to traditional pasteurization while preserving physicochemical, sensory, and microbiological quality. It also enhanced bioactive compounds, proving its potential as an efficient and quality‐preserving juice processing technology.

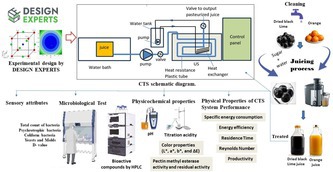

## 1 Introduction

Citrus juices are considered a vital source for meeting the daily requirements of fruits and vegetables due to their optimal content of bioactive compounds, such as antioxidants such as vitamin C carotenoids (Arilla et al. 2022), and flavanones (Saini et al. 2022). They also serve as an important source of minerals, sugars, water, dietary fiber, and other nutrients and organic compounds (Wang et al. 2023). However, fruit juices can also provide a favorable environment for microbial growth and proliferation, which can lead to spoilage. Therefore, controlling the microbial load is crucial for extending shelf life (Ercan and Soysal 2013). Traditional thermal processing is an effective technique for microbial inactivation. Nevertheless, excessive thermal treatment can cause undesirable changes in the functional properties of foods, such as the degradation of vitamins and the development of off‐flavors (Narra et al. 2024). Thermal processing methods are often expensive and environmentally unfriendly, exhibiting a significant environmental burden either directly through fossil fuel combustion or indirectly via the use of heating elements to generate and transfer heat to food products (Dhenge et al. 2022). These processes also demand long processing times and high energy inputs. Therefore, the world is now moving toward producing healthy foods with properties similar to fresh foods (Martinho et al. 2022), by developing effective and innovative non‐thermal technologies that ensure the preservation of the properties of the food substance (Chiozzi et al. 2022). Among these emerging technologies, Thermosonication (TS) has gained attention as a promising method that combines ultrasound (US) and mild heat (typically 37°C–75°C). TS is increasingly accepted by both consumers and food producers due to its ability to enhance food quality and safety while preserving fresh‐like characteristics. Additionally, TS significantly minimizes detrimental changes in sensory attributes and is considered an eco‐friendly, cost‐effective, and time‐efficient process, making it a viable alternative for food preservation (Adebo et al. 2021). Furthermore, TS has demonstrated efficacy in inactivating both microorganisms and enzymes (Chávez‐Martínez et al. 2020), and is now recognized as an alternative to conventional thermal processing due to its high potential in improving the functional and biochemical properties of foods (Altemimi et al. 2017). Das et al. (2024) reported that TS significantly enhanced the sensory qualities of Sohphie juice and facilitated the release of various bioactive compounds, including phenolics, flavonoids, ascorbic acid, anthocyanins, and carotenoids, in comparison to untreated and conventionally pasteurized samples. Previous studies have elucidated that US energy is generated by converting electrical energy into mechanical energy using a Langevin piezoelectric transducer. During liquid food processing, the ultrasound waves propagate through the medium, inducing pressure fluctuations that result in bubble formation. These bubbles subsequently collapse with great intensity during compression cycles, generating localized zones of high temperature and pressure, a phenomenon known as acoustic cavitation (Rani et al. 2025). The main challenge of current TS systems is determining the optimal processing parameters. Long processing times, low productivity, and high energy consumption (depending on the experimental inputs) are other challenges facing this emerging technology. Due to the scarcity of continuous flow pelvic TS systems, all currently designed devices operate either in the probe mode or in the continuous flow mode (Deshpande and Walsh 2021; Deshpande and Walsh 2020), or using pelvic ultrasound devices in batch recirculation mode (Demir and Kılınç 2019). Therefore, this study aimed to develop a new continuous thermoacoustic processing (CTS) system by modifying the settings of the existing batch system. The goal is to improve production efficiency, reduce energy consumption, and evaluate the system's performance. Additionally, the study investigates the effects of the CTS process on the physical, chemical, microbial, and sensory properties of the processed juices, and seeks to optimize processing conditions using response surface methodology.

## 2 Materials and Methods

### 2.1 Chemicals and Reagents

Pectin and phenolphthalein were purchased from Fluka AG (Buchs, Switzerland). Sodium hydroxide (NaOH) and sodium bicarbonate (NaHCO_3_) were obtained from Central Drug House (India). Ascorbic acid and sodium chloride (NaCl) were supplied by TM Media (India), while acetic acid was sourced from B.D.H. (England). All microbiological culture media used in this study were procured from Himedia (Mumbai, India). Metaphosphoric acid (HPO_3_), 2,6‐dichlorophenolindophenol (DCIP), trifluoroacetic acid (TFA), acetonitrile, and all analytical standards including gallic acid, luteolin, hesperidin, ferulic acid, naringenin, apigenin, and rutin were obtained from Sigma‐Aldrich (Germany). All standard compounds were of chromatographic grade, while other reagents used were of analytical grade.

### 2.2 Preparation of Orange Juice (OJ)

Ripe *Citrus sinensis var. Valencia* fruits were sourced from local markets in Basra, Iraq. OJ was extracted following the method of Sun et al. (2022), with minor modifications. After thorough washing, the fruits were peeled, sliced, and processed using an electric juicer (Model JCR‐6135, Turkey). The juice was filtered through sterile muslin cloth to remove pulp and particulates, then transferred to sterilized containers and stored at 5°C ± 2°C until analysis.

### 2.3 Preparation of Dried Black Lime Juice (DBLJ)

Seedless dried black lime ( *Citrus aurantifolia* L.), locally known as Guatemalan lemon or key lime, was obtained from markets in Basrah, Iraq, ensuring freedom from mechanical damage. Juice preparation followed the method of Aboud et al. (2020), with slight modifications. Briefly, 500 g of dried black lime were ground using a high‐speed electric grinder (850 W, 220 V, 50 Hz). The resulting powder was mixed with 10 L of water and 1550 g of sugar, then refrigerated at 5°C ± 2°C for 24 h. The mixture was filtered through sterilized muslin cloth, and the extract was stored at 5°C in sterile containers until further analysis.

### 2.4 Traditional Pasteurization

Traditional pasteurization (TP) was applied to OJ and DBLJ using a German‐made water bath (Type 1003, no. 1614811K, Power 1.5 kW). The juice was placed in a 4‐L stainless steel vessel (type 316) and heated in the water bath to 90°C for 60 s, following the method of Oladunjoye and Awani‐Aguma (2023). The juice was then immediately cooled by immersion of the container in an ice‐water bath.

### 2.5 Continuous Thermosonication System

**FIGURE 1:**
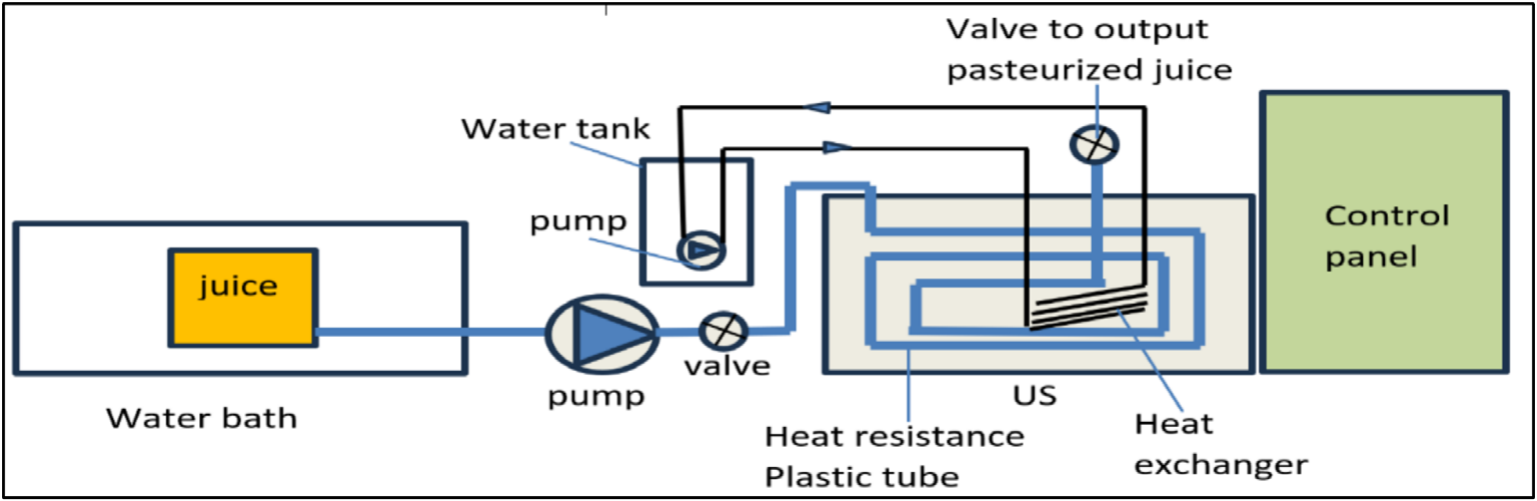
CTS schematic diagram.

The CTS system is shown in Figure 1, Figures S1 and S2. It was designed and manufactured in the Food Engineering Laboratory, Department of Food Science, College of Agriculture, University of Basrah, Basrah, Iraq. A Korean basal US, 40 kHz, 20‐stage batch US (LUC‐405 Model, Voltage: 220 V/50 Hz) was developed to a continuous US system via using a water bath (heating unit) used to raise the juice temperature to the processing temperature before the entrance of the juice to the basal US. A centrifugal pump was used to transfer the heated juice from the heating unit to the 40 m of heat‐resistant plastic tube put into the US basal. The purpose of this tube was to circulate heated juice in the basal of US for treating it by US. To control the temperature rise during juice treatment by US, a heat exchanger was utilized. The system was also provided with a control panel, and electrical power supply unit. More details of the CTS system are presented in Figures S1 and S2 with an in‐depth explanation. The CTS application conditions included three power levels (1.676, 1.72, and 1.764) kW, temperature (30°C, 40°C, and 50°C), and mass flow rates (0.0142, 0.0079, and 0.0016) kg/s. The MFR was calculated by measuring the juice weight (kg) at a specific time unit (s). The experiments were repeated three times.

### 2.6 Studied Characteristics of the CTS Device Performance

#### 2.6.1 Power (kW)

The Power was calculated for both the US device and the water bath (WB) according to the following equation:

(1) $$P_{\mathrm{WB},\mathrm{US}}=V\;I\;;\;\left(1W=\frac{J}{\sec}\;\right)$$

where *I*: electric current (*A*), *V*: voltage (*V*). The voltage and current were measured by a digital meter in the electrical power supply unit, while the total power was calculated according to the following equation.

(2) $$\text{Power}=P_{\mathrm{WB}}+P_{\mathrm{US}}$$

where: $P_{\mathrm{WB}}$ is the power of the water bath, $P_{\mathrm{US}}$ is power of the US device. The power is converted to kW by dividing it by 1000.

#### 2.6.2 Specific Energy Consumption (kJ/kg)

The specific energy consumption (SEC) was calculated according to the following equation:

(3) $$\mathrm{SEC}={\mathrm{SEC}}_{\mathrm{WB}}+{\mathrm{SEC}}_{\mathrm{US}}$$

where SEC_US_: Specific energy consumption of the US device, ${\mathrm{SEC}}_{\mathrm{WB}}:$ Specific energy consumption of the water bath. The SEC for both the US device and the water bath was calculated according to the following equation described by Cheaib et al. (2018).

(4) SEC=Pt/M

*P*: input power (kW = kJ/s), *t*: time (s), *M*: Juicy mass (kg).

#### 2.6.3 Energy Efficiency (%)

Energy efficiency (EE) was calculated according to Equation (5) and described by (Al‐Hilphy et al. 2020).

(5) $$\mathrm{EE}\%=\;\frac{Q_{0}}{Q_{\text{in}}}\times 100$$

where $Q_{0}$: output power (kW = KJ/s), $Q_{\text{in}}$: input power (kW = KJ/s).

#### 2.6.4 Juice Residence Time (Min)

The juice residence time (RT) within the system was calculated according to Alsaedi et al. (2023).

(6) $$\mathrm{RT}=\frac{\mathrm{\rho A}L_{d}}{\mathrm{MFR}}$$

where *ρ*: juice density (kg/m^3^), A: is the area (m^2^), $L_{d}$: pipe length (m), MFR: ˙mass flow rate of juice (kg/s), the RT is converted to min by dividing it by 60.

#### 2.6.5 Reynolds Number

The Reynolds number (Re) of the juice inside the CTS system was calculated according to Singh et al. (2025).

(7) Re=4MFRπμD

μ; juice viscosity (kg/m s. = Pa. s), *D*: pipe diameter (m).

#### 2.6.6 Productivity (L/h)

The Productivity (Pr) was calculated by calculating the amount of juice produced after treatment per unit of time.

#### 2.6.7 Continuous‐Use Simulation for Temperature and Acoustic Intensity of CTS

A continuous‐use simulation for temperature and acoustic intensity of CTS was executed by MATLAB R2014a. The code and simulation of data were presented in Figure S6.

### 2.7 Quality Tests for Juice

#### 2.7.1 Physicochemical Tests

The pH was determined for all treatments using an EMCO pH‐meter (Singapore‐made S/N27657236). Before starting the tests, buffer solutions with pH 4, 7, and 10 were used to calibrate the pH‐meter. The titratable acidity was determined according to Yildiz et al. (2019) by placing 10 mL of juice sample in a clean beaker, adding 3–5 drops of 1% phenolphthalein indicator, and titrating with 0.1 M NaOH until a pink color was obtained at pH 1 ± 8.2. The acidity was expressed as a percentage of citric acid content according to the following equation:

(8) $$\begin{array}{c} \text{Titrable acidity}\;\left(\%\text{citric acid}\right)\\ \;=\;\frac{\;\mathrm{mL}\;\text{of NaOH}\;\left(0.1\;N\right)\times 0.064\;}{\text{volume of sample}\;\left(\mathrm{mL}\right)}\times 100 \end{array}$$

Color characteristics were analyzed using image processing techniques as described by Al‐Hilphy et al. (2020), with slight modifications. In brief, the juice sample was placed in a Petri dish, and images were captured inside a wooden imaging box equipped with a high‐resolution camera (720 pixels, IP67 Endoscope, Mileseey, China) and four LED lights. Two of the lights were positioned at a 45° angle relative to the camera lens, while the other two were oriented perpendicularly to the sample surface. After capturing the digital images, ImageJ software (version 1.54q, National Institutes of Health, USA) was used to extract the L*, a*, and b* color parameters in three replicates (Annisa et al. 2023; Bornowski et al. 2020). The total color difference (∆E) was calculated according to the method described by Wang et al. (2025) using the following equation:

(9) $$∆E=\sqrt{{\left(L_{o}^{*}-L^{*}\right)}^{2}+{\left(a_{o}^{*}-a^{*}\right)}^{2}+{\left(b_{o}^{*}-b^{*}\right)}^{2}}$$

where L* represents lightness (ranging from white = 100 to black = 0), a* indicates the red‐green coordinate (positive values toward red, negative toward green), and b* denotes the yellow‐blue coordinate (positive values toward yellow, negative toward blue) for the treated juice samples. In contrast, $L_{o}^{*}$, $a_{o}^{*}$, and $b_{o}^{*}$ refer to the corresponding color parameters of the untreated (fresh) juice sample.

#### 2.7.2 Pectin Methyl Esterase Activity and Residual Activity

The activity of the enzyme pectin methylesterase (PME) was determined according to the method described by Kimball (1999) and modified by Al‐Hilphy et al. (2023). Briefly, one liter of a 1% pectin salt solution was prepared by dissolving 10 g of pectin and 15.3 g of NaCl in distilled water and adjusting the final volume to 1000 mL. Additionally, two separate sodium hydroxide (NaOH) solutions with concentrations of 2 M and 0.05 M were prepared. Subsequently, 10 mL of juice was mixed with 40 mL of the 1% pectin solution in a 100 mL beaker. This beaker was then placed into a larger 250 mL beaker filled with an appropriate amount of distilled water to act as a water bath. Both beakers were placed on a magnetic stirring hot plate set to 30°C, and the temperature was monitored using a mercury thermometer. Drops of 2 M NaOH solution were added to the sample pectin mixture until the pH reached approximately 7.6–7.8. Then, 0.1 mL of 0.05 M NaOH solution was added to the mixture, and a stopwatch was immediately started. The time required for the pH to return to its original value prior to the NaOH addition was recorded. PME activity (unit/mL) was calculated using the following equation:

(10) $$\begin{array}{c} \text{Enzyme activity of}\;\mathrm{PME}\left(\frac{\text{unit}}{\mathrm{mL}}\right)\\ \;=\frac{\text{NaOH}\;\left(0.05N\right)\times 0.1\;\mathrm{mL}\;\text{NaOH}\;\left(0.05N\right)}{10\;\mathrm{mL}\;\text{of sample}\times \text{time}\;\left(\text{minute}\right)} \end{array}$$

(11) $$\text{Residual}\;\mathrm{PME}\;\text{activity}\;\left(\mathrm{RA},\%\right)=\frac{A_{t}}{A_{0}}\times 100$$

The residual activity percentage was calculated according to the following equation.

Where $A_{t}$ represents the enzymatic activity of the treated juice and $A_{0}$ denotes the enzymatic activity of the fresh juice.

#### 2.7.3 Microbiological Tests

Total count of bacteria (TCB), psychrotrophic bacteria (Ps), coliform bacteria (CB), yeasts and molds (Y&M) were conducted using the plate count method to determine the number of microorganisms in the juice samples. Briefly, 1 mL of the juice sample was added to 9 mL of peptone water containing 0.1% peptone. A series of decimal dilutions were prepared using sterile pipettes and test tubes to estimate the TCB, Ps, CB, and Y&M. mL of each dilution was transferred to sterile petri dishes and then Nutrient Agar prepared by Himedia India according to the manufacturer's recommendations was poured onto the medium. Three replicates were used for each dilution. The plates were incubated at 37°C for 24–48 h to determine the TCB (Jabbar et al. 2015), while incubation for 7–10 days at 5°C ± 2°C was used to estimate Ps colonies (Jay et al. 2003). For CB enumeration, the same procedure was followed, except MacConkey Agar medium was used in place of Nutrient Agar, and the plates were incubated at 37°C for 24–48 h (Islam et al. 2025). Yeasts and molds (Y&M) were quantified using potato dextrose agar, incubated at 25°C–28°C for 3–5 days (Abedelmaksoud et al. 2019).

#### 2.7.4 Curve Fitting by Weibull Model of TBC

Plotting the logarithm of survivors versus treatment duration yielded survival curves after plate counts were converted to Log10 values. An equation based on the Weibull distribution was used to fit survival curves as follows (Mafart et al. 2002):

(12) $$\log_{10}\left(\frac{N}{N_{o}}\right)=-{\left[\frac{t}{\delta }\right]}^{p}$$

where $N_{o}$ is the initial bacterial population, *N* is the surviving population at time *t*, *δ* is the scale parameter, representing the time needed for the first decimal reduction (time for 90% reduction), *p* is the shape parameter.

#### 2.7.5 Decimal Reduction Time (*D*‐Value)

The time required to reduce 90% of the microorganisms was calculated according to the following equation described by (Dash et al. 2022).

(13) $$D-\text{value}=\frac{\mathrm{RT}}{\;{\text{logN}}_{\mathrm{oj}}-{\text{logN}}_{j}}$$

*N*_oj_: Initial microbial count (CFU/mL), *N*_j_: Final microbial count (CFU/mL), RT: Retention time of the juice in the system (min).

#### 2.7.6 Estimation of Some Bioactive Compounds Using Reversed Phase‐High‐Performance Liquid Chromatography (RP‐HPLC)

The analysis was performed using (RP‐HPLC) on a SYKAM HPLC system (Germany) equipped with a C18–ODS column (250 × 4.6 mm, 5 μm). Samples were injected into the system at a volume of 100 μL with a flow rate of 1 mL/min. The mobile phase consisted of 95% Acetonitrile +0.01% Trifluoroacetic acid (solvent A) and 5% Acetonitrile +0.01% Trifluoroacetic acid (solvent B). The gradient program was as follows: 10% A from 0 to 5 min, 25% A from 5 to 7 min, 40% A from 7 to 13 min, followed by a return to the initial conditions. The phenolic compounds were detected using a UV–visible detector at 278 nm. The phenolic compounds were identified based on retention times and compared with standard compounds prepared at a concentration of 5 ppm for each phenolic compound: Luteolin, Hesperidin, Ferulic acid, Naringenin, Apigenin, Rutin, p‐Coumaric acid, and Gallic acid.

#### 2.7.7 Sensory Evaluation

The sensory evaluation was conducted according to the method outlined by Kalsi, Singh, Alam, and Bhatia (2023), with some modifications to the evaluation form. The test was carried out in the Food Engineering Laboratories under standard lighting conditions at a temperature of 25°C. Fifteen experts, including professors and PhD students in the Department of Food Sciences, participated in the study, including nine females and six males. Participants' ages ranged from 23 to 35 years (60%) and from 36 to 60 years (40%). The samples were divided into three groups, each represented by a code corresponding to the treatment type. Panelists were instructed to cleanse their palates with drinking water before and after the sensory evaluation of the juice samples, with a set rest period to ensure optimal evaluation performance. The juice samples were evaluated in terms of appearance, color, odor, taste, and overall acceptability using a nine‐point hedonic scale. The sensory scores recorded on the evaluation form were as follows: 9 = Extremely liked, 8 = Liked very much, 7 = Liked moderately, 6 = Liked slightly, 5 = Neither liked nor disliked, 4 = Disliked slightly, 3 = Disliked moderately, 2 = Disliked very much, 1 = Not acceptable at all.

### 2.8 Process Improvement and Data Modeling

All independent variables were selected based on preliminary experiments conducted for this purpose. The Design Expert software, version 13 (Stat‐Ease INC., USA), was used to determine the factorial parameters and study the effect of three independent factors: power (1.676, 1.72, 1.764) kW, temperature (30°C, 40°C, 50°C), and MFR (0.0142, 0.0079, 0.0016) kg/s, as well as their interactions, on the studied attributes for predicting empirical equations. The independent variables were coded as (−1, 0, +1), representing the lowest, medium, and highest values, respectively. A total of 20 treatments were randomly distributed, including 6 central points. In addition, optimal values for the independent variables were determined using the Response Surface Methodology (RSM) with Central Composite Design (CCD). The quadratic polynomial regression model (Equation 14) was used to predict the studied attributes.

(14) $$Y=\alpha_{o}+\sum_{i=1}^{k}\alpha_{i}X_{i}+\sum_{i=1}^{k}\alpha_{\mathrm{ii}}X_{i}^{2}+\sum \sum_{i<j=1}^{k-1}\alpha_{\mathrm{ij}}X_{i}X_{j}$$

*Y*: Response (attributes), *k*: Number of factors, $\alpha_{o}$: Constant term, $\alpha_{i}$: Coefficient related to the linear term in the equation, $\alpha_{\mathrm{ii}}$: Coefficient associated with the nonlinear (quadratic) term in the equation, $\alpha_{\mathrm{ij}}$: Coefficient corresponding to the interaction term in the equation, $X_{i},X_{j}$: Independent variables, where *i* and *j* represent the indices of the factors.

### 2.9 Statistical Analysis

The Design Expert software, version 13 (Stat‐Ease Inc., United States), was utilized to perform the analysis of variance (ANOVA). Additionally, SPSS software, version 21, was employed to analyze the data using a one‐way experimental design to determine the significance among predicted and experimental data, conventional pasteurization, and fresh samples. The least significant difference (LSD) test at a significance level of *p* < 0.05 was applied to compare the means of the different treatments.

## 3 Results and Discussion

### 3.1 Physical Properties of CTS System Performance

#### 3.1.1 Specific Energy Consumption (kJ/kg)

**FIGURE 2:**
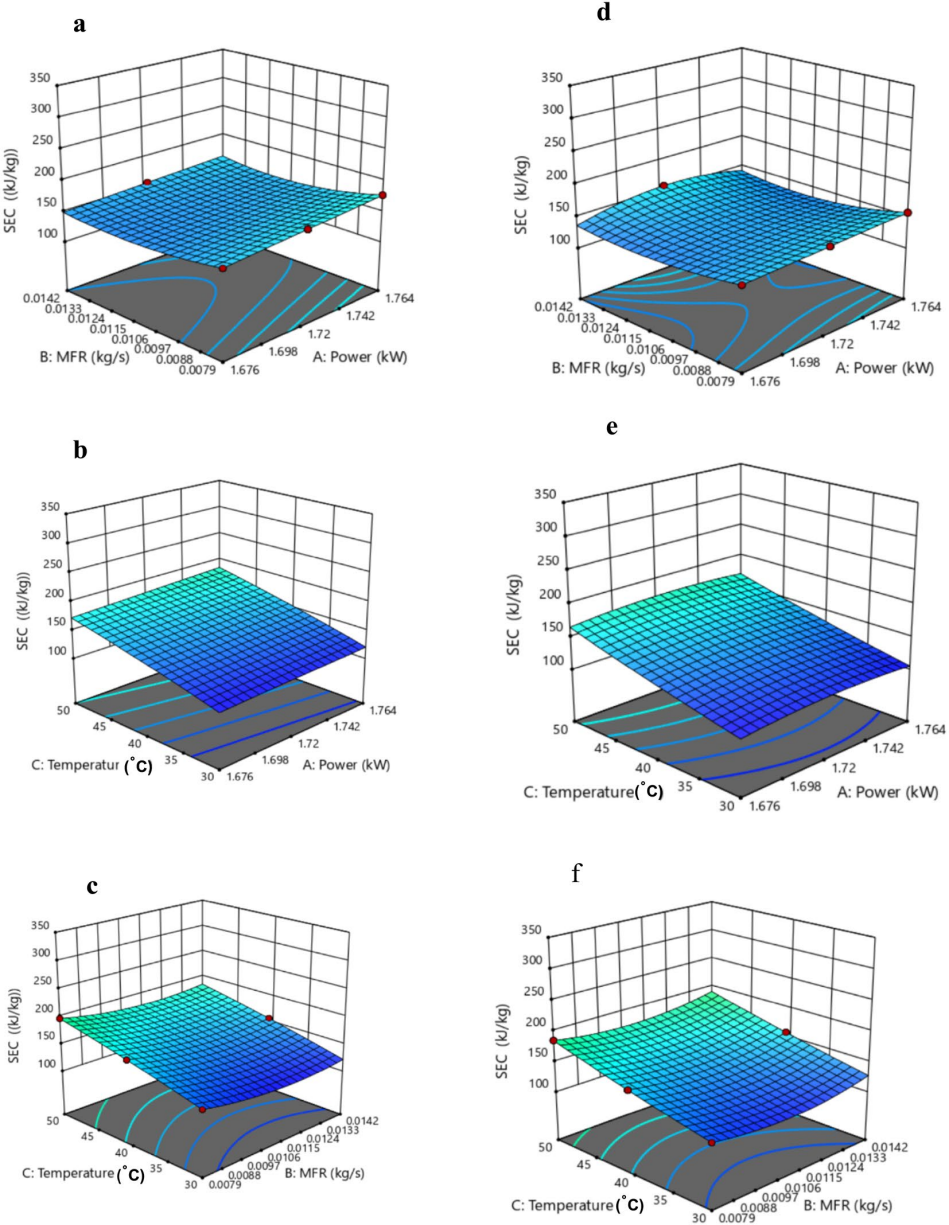
Response surface plots showing the effect of independent variables on SEC (kJ/kg): (a–c) Interactions between independent factors for OJ; (d–f) interactions between independent factors for DBLJ.

Table 1 presents the CCD matrix illustrating the effect of power, MFR, and temperature on the SEC values of OJ and DBLJ after treatment with CTS. The results indicated that the SEC values ranged from 104.87 to 312.38 kJ/kg for OJ and from 105.99 to 322.5 kJ/kg for DBLJ. The highest and lowest SEC values for both OJ and DBLJ were observed in Runs 3 and 9, respectively. According to the ANOVA results presented in Tables S1 and S2, the quadratic model (QM) for OJ and the quartic model (QuM) for DBLJ showed a statistically significant effect (*p* < 0.05) of power and MFR. In contrast, the lack of fit (LOF) was not statistically significant (*p* > 0.05), suggesting an adequate model fit. Furthermore, a significant interaction (*p* < 0.05) between power and MFR was observed, while other interaction terms did not have a significant impact (*p* > 0.05) on SEC values for OJ and DBLJ, according to the fit statistical indicators such as *R* ^2^ (0.99, 0.99) adjusted *R* ^2^ (0.99, 0.99), and Adeq Precision (11702.12, 61.5715) for OJ and DBLJ, respectively, the QM and QuM can be used for predicting SEC values, the regression coefficients for the QM and QuM models are provided in Table S3. The 3D surface plots generated using RSM, as shown in Figure 2, indicate that SEC values decreased with increasing MFR and decreasing power and temperature. This trend can be attributed to the influence of these parameters on the time required to reach the target processing temperature higher temperatures demand longer heating durations. These results were consistent with the findings of Al‐Hilphy et al. (2024), as researchers noted a decrease in SES values with decreasing power and treatment time. For example, a reduction in power from 1.72 to 1.676 kW at an MFR of 0.0079 kg/s and temperature of 40°C led to a decrease in SEC from 150.07 and 151.14 kJ/kg to 144.41 and 145.57 kJ/kg for OJ and DBLJ, respectively. Table 2 presents the SEC values under optimized CTS conditions for OJ (CTS‐OJ: power = 1.762 kW, MFR = 0.002 kg/s, temperature = 45.13°C) and DBLJ (CTS‐DBLJ: power = 1.762 kW, MFR = 0.002 kg/s, temperature = 46.68°C) in comparison to TP. Optimization results revealed no significant differences (*p* > 0.05) between the experimental and predicted SEC values. However, the SEC values for CTS‐OJ and CTS‐DBLJ were significantly lower (*p* < 0.05) than those of TP by 29.71% and 30.51%, respectively. This reduction is attributed to the shorter time required to reach the target processing temperature and the lower overall energy input during CTS treatment.

**TABLE 1:** CCD matrix for the effects of power, MFR, and temperature on the physical properties of CTS performance of OJ and DBLJ.

RUN	Independent variables						Dependent variables						
	OJ						DBLJ						
	P	MFR	T	SEC	EE	RT	Re	Pr	SEC	EE	RT	Re	Pr
1	1.72	0.0142	40	137.67	82.12	0.16	5835.22	42.96	156.88	80.6	0.16	5320.3	42.57
2	1.72	0.0079	50	175.22	90.36	0.28	3068.2	35.13	185.35	83.27	0.28	2875.3	32.95
3	1.676	0.0142	30	104.87	74.25	0.16	5911.49	56.25	105.99	72.67	0.16	5351.8	55.58
4	1.72	0.0079	40	150.02	81.82	0.28	3144.9	42.01	151.15	80.47	0.28	2859	41.63
5	1.764	0.0142	30	111.07	70.54	0.16	5911.49	56.24	112.19	69.03	0.16	5320.3	55.58
6	1.676	0.0016	50	257.38	92.91	1.39	649.114	30.21	267.5	86.11	1.41	569.33	28.55
7	1.72	0.0079	30	120.32	72.34	0.28	3377.08	54.64	121.45	70.77	0.28	2942.6	54
8	1.764	0.0016	30	257.48	70.52	1.39	661.759	43.6	258.6	69.01	1.4	599.48	43.15
9	1.764	0.0016	50	312.38	88.11	1.4	621.407	30.21	322.5	81.16	1.41	579.04	28.55
10	1.764	0.0142	50	165.97	88.26	0.16	5617.76	35.78	176.09	81.91	0.16	5168.3	33.53
11	1.72	0.0079	40	150.02	81.89	0.28	3125.37	42.01	151.15	80.37	0.29	3013.1	41.63
12	1.72	0.0079	40	150.02	82.14	0.28	3288.79	42.01	151.15	80.59	0.28	2795.5	41.64
13	1.72	0.0079	40	150.02	81.82	0.28	3125.37	42.01	151.15	80.47	0.28	2959.9	41.63
14	1.72	0.0016	40	259.68	81.9	1.39	632.987	35.17	260.8	80.62	1.4	599.48	34.87
15	1.676	0.0142	50	159.77	92.98	0.16	5797.81	35.78	169.89	86.18	0.16	5109.9	33.53
16	1.764	0.0079	40	155.59	80.01	0.28	3144.9	42.01	156.72	78.57	0.28	2942.6	41.64
17	1.72	0.0079	40	150.02	81.93	0.28	3087.02	42.01	151.15	80.61	0.28	2842.9	41.64
18	1.676	0.0016	30	202.48	74.25	1.39	666.084	43.61	203.6	72.67	1.4	606.61	43.15
19	1.72	0.0079	40	150.02	82.13	0.28	3267.43	42.01	151.15	80.64	0.28	2959.9	41.64
20	1.676	0.0079	40	144.45	84.28	0.28	3267.43	42.01	145.58	82.73	0.28	2977.4	41.64

Abbreviations: DBLJ, dried black lime juice; EE, energy efficiency (%); MFR, mass flow rate (kg/s); OJ, orange juice; P, power (kW); Pr, productivity (L/h); Re, reynolds number; RT, juice residence time in the device (min); SEC, specific energy consumption (kJ/kg); T, temperature (°C).

**TABLE 2:** Results of the optimization process for the predicted and experimental responses of OJ and DBLJ treated with CTS‐OJ and CTS‐DBLJ compared to TP and the fresh sample.

Dependent variables	OJ				DBLJ			
	CTS‐OJ		TP	Fresh	CTS‐DBLJ		TP	Fresh
	Experimental	Predicted			Experimental	Predicted		
SEC (kJ/kg)	273.30 ± 2.65^b^	276.43 ± 0.10^b^	388.8 ± 4.65^a^	—	280.12 ± 8.59^b^	275.15 ± 0.035^b^	403.13 ± 7.49^a^	—
EE (%)	83.16 ± 1.59^a^	84.60 ± 0.06^a^	77.94 ± 0.76^b^	—	81.88 ± 1.85^a^	81.47 ± 0.04^a^	75.91 ± 3.33^b^	—
RT (min)	1.24 ± 0.032^a^	1.23 ± 0.035^a^	1 ± 0.00^b^	—	1.3 ± 0.02^a^	1.31 ± 0.0001^a^	1 ± 0.00^b^	—
Re	770.12 ± 20.58^a^	772.75 ± 0.1^a^	—	—	721.27 ± 25.59^a^	719.64 ± 0.04^a^	—	—
Pr (L/h)	32.45 ± 0.52^a^	32.09 ± 0.057^a^	13.13 ± 0.14^b^	—	31.19 ± 1.52^a^	30.90 ± 0.08^b^	12.63 ± 0.27^c^	—
pH	4.33 ± 0.006^a^	4.33 ± 0.003^a^	4.26 ± 0.01^b^	4.33 ± 0.01^a^	3.36 ± 0.006^a^	3.37 ± 0.004^a^	3.28 ± 0.01^b^	3.36 ± 0.01^a^
T.A (%)	0.787 ± 0.0065^b^	0.793 ± 0.0062^b^	0.866 ± 0.01^a^	0.787 ± 0.013^b^	0.992 ± 0.006^b^	0.995 ± 0.006^b^	1.105 ± 0.004^a^	0.971 ± 0.005^c^
L*	73.64 ± 0.08^a^	73.68 ± 0.006^a^	71.45 ± 0.26^c^	74.53 ± 0.19^b^	73.11 ± 0.23^a^	72.98 ± 0.11^a^	70.76 ± 0.57^c^	73.79 ± 0.17^b^
a*	−4.70 ± 0.02^b^	−4.72 ± 0.053^b^	−6.7 ± 0.18^c^	−4.52 ± 0.029^a^	16.11 ± 0.03^b^	16.04 ± 0.02^b^	14.56 ± 0.37^c^	16.50 ± 0.04^a^
b*	70.55 ± 0.087^b^	70.53 ± 0.03^b^	68.23 ± 0.97^c^	71.50 ± 0.16^a^	39.82 ± 0.45^b^	39.84 ± 0.046^b^	37.98 ± 0.45^c^	40.70 ± 0.63^a^
ΔE	1.23 ± 0.10^b^	1.24 ± 0.10^b^	5.01 ± 0.83^a^	—	1.19 ± 0.45^b^	1.16 ± 0.08b	4.55 ± 0.23^a^	—
PEM (unit/mL)	0.0000119 ± 0.0000002^b^	0.0000118 ± 0.00000008^b^	0.0000111 ± 0.0000001^b^	0.000159 ± 0.000004^a^	0.0000075 ± 0.0000001^b^	0.00000746 ± 0.0000001^b^	0.00000694 ± 0.00000005^b^	0.0001167 ± 0.000005^a^
RA (%)	7.48 ± 0.11^b^	7.44 ± 0.04^b^	6.98 ± 0.075^a^	—	6.42 ± 0.074^b^	6.39 ± 0.082^b^	5.95 ± 0.04^a^	—
TBC (log cfu/mL)	ND^c^	0.31 ± 0.002^b^	ND^c^	2.75 ± 0.115^a^	ND^c^	0.401 ± 0.008^b^	ND^c^	2.96 ± 0.75^a^
*D*‐value (min)	0.407 ± 0.01^a^	0.425 ± 0.004^a^	0.365 ± 0.01^b^	—	0.380 ± 0.01^b^	0.391 ± 0.0006^a^	0.338 ± 0.009^c^	—

*Note:* CTS‐OJ: continuous thermosonication at optimum conditions for OJ (power: 1.764 kW, MFR: 0.002 kg/s, temperature: 45.13°C), CTS‐DBLJ: continuous thermosonication at optimum conditions for DBLJ (power: 1.764 kW, MFR: 0.002 kg/s, temperature: 46.68°C), TP: conventional pasteurization (90°C for 60 s), pH: hydrogen permeability, L*: clarity (black/white), a*: (red/green), b*: (yellow/blue), ΔE: total color change, ND: not detected (< 1 CFU/mL), *D*‐value: time required for 90% destruction of microorganisms. Different letters in the same row refer to significant differences at a level of 0.05.

Abbreviations: PME, pectin methylesterase; RA, residual activity; TA, titration acidity; TBC, total bacterial count.

#### 3.1.2 Energy Efficiency (%)

**FIGURE 3:**
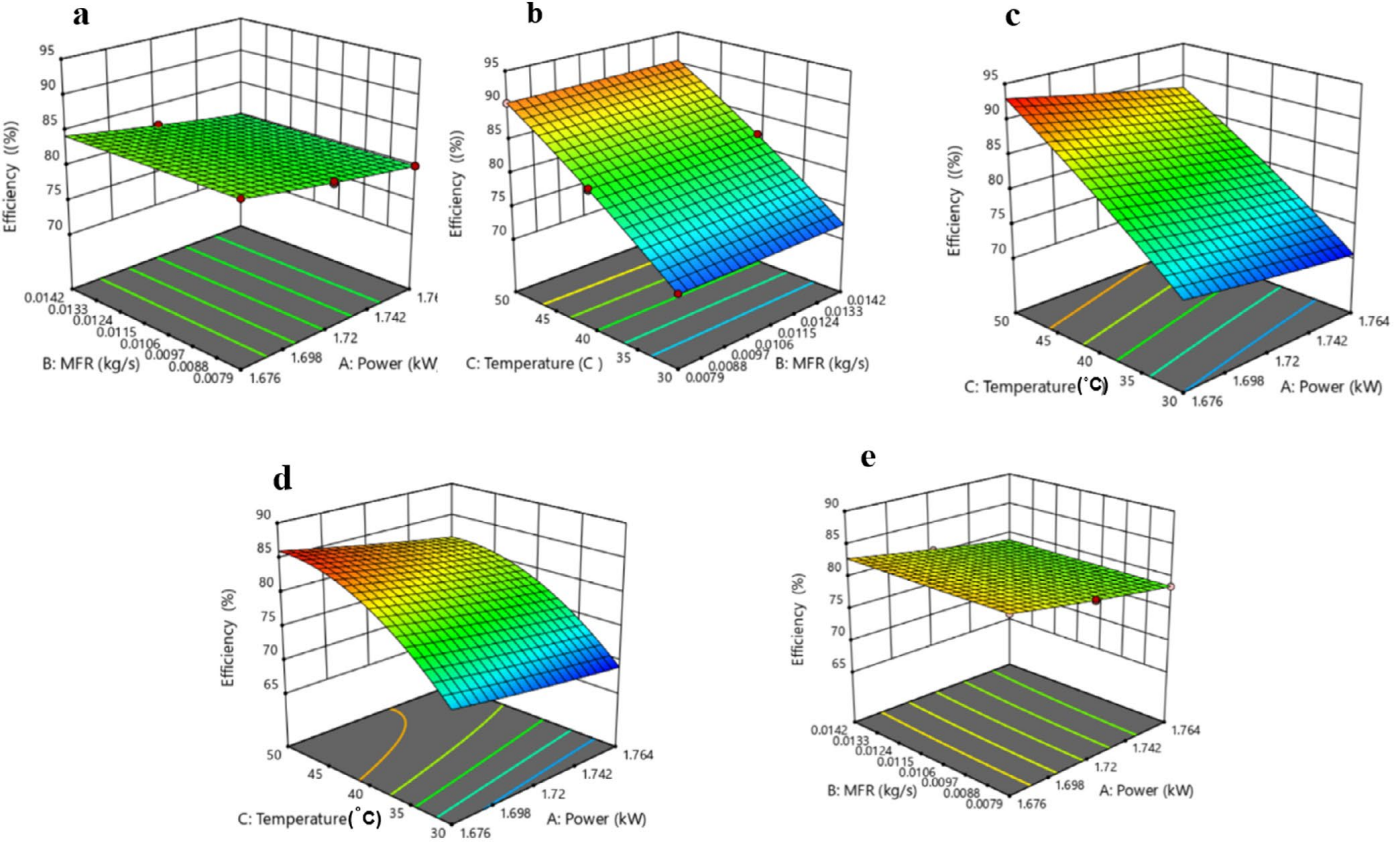
Response surface plots showing the effect of independent variables on EE (%). (a–c) Interactions between independent factors for OJ. (d, e) Interactions between independent factors for DBLJ.

According to the results presented in Table 1, the EE% values for OJ and DBLJ ranged between 70.52%–92.98% and 69.01%–86.18%, respectively. The highest and lowest values for both OJ and DBLJ were observed in Runs 15 and 8, respectively. As shown in Tables S1 and S2, the QM for OJ and DBLJ indicated that power, temperature, temperature square, and the interaction between power and temperature had a significant effect (*p* < 0.05) on EE% values. In contrast, LOF and other factors and interactions exhibited no significant effects (*p* > 0.05) on the EE% of the treated juices. Statistical indicators such as *R* ^2^ = 0.99, Adjusted *R* ^2^ = 0.99, and Predicted *R* ^2^ = 0.99 for both OJ and DBLJ confirm the adequacy of the QM model in predicting EE% values, with regression coefficients presented in Table S3. The three‐dimensional response surface plots (Figure 3) generated by RSM illustrate that EE% increased concurrently with decreasing power and increasing temperature, while MFR showed no significant impact on EE%. For instance, decreasing power from 1.764 to 1.676 kW at MFR of 0.0142 kg/s and temperature 30°C led to an increase in EE% from 70.58% and 69.13% to 74.28% and 72.61% for OJ and DBLJ, respectively. This improvement is attributed to the reduced energy consumption rates. To establish a mathematical model with optimal fit to the experimental data, the interaction effect between MFR and temperature for DBLJ was excluded due to its lack of statistical significance (*p* > 0.05), which would otherwise weaken the model. The optimization results in Table 2 revealed no significant difference (p > 0.05) between the experimental and predicted EE% values, indicating the reliability of the model. Furthermore, the EE% values for CTS‐OJ and CTS‐DBLJ were significantly higher (*p* < 0.05) than those obtained by TP, by 6.70% and 7.86%, respectively. To our knowledge, there are no previous studies that have examined the energy efficiency of TS systems for juice pasteurization. Therefore, scalability and comparisons are challenging. The wide variation in processing conditions and the diversity of ultrasonic devices present another challenge to obtaining standardized data on appropriate energy inputs. Numerous global efforts are currently underway to standardize optimal processing conditions across emerging technologies. Conversely, the current study could provide a more comprehensive database and facilitate comparison of the results of different studies. In this context, we note that the QM developed in this study could provide a solid foundation for implementing these improvements more widely in industry. The optimization results obtained under optimal conditions indicate an improvement in the energy efficiency of the CTS system compared to the TP system. This improvement not only contributes to reducing energy and current consumption but also reduces the time required to reach processing temperature. This, in turn, contributes to increased productivity, lower costs, and thus increases the overall efficiency of the CTS system. Therefore, applying these improvements in industrial production environments can achieve similar positive results.

#### 3.1.3 Residence Time (Min)

Determining the RT of liquids in the pasteurization unit is a critical parameter in the design of food processing systems due to its direct influence on the extent of physicochemical changes that occur during treatment. The results indicated that RT values ranged from 0.157 to 1.39 min for OJ and from 0.158 to 1.40 min for DBLJ. The highest RT was recorded at an MFR of 0.0016 kg/s, while the lowest RT occurred at an MFR of 0.0142 kg/s for both OJ and DBLJ, which is attributed to the decrease in juice velocity with lower MFR (Table 1). Furthermore, as shown in Tables S1 and S2, the Reduced Cubic Model (RCM) for OJ and the QM for DBLJ, along with MFR, MFR^2^, and the interaction between Tand MFR, had a statistically significant effect (*p* < 0.05) on RT values for both juice types. Additionally, the three‐way interaction among the studied factors, as well as the interactions between temperature and power square, and between MFR and power square, had a significant effect on RT values for OJ only. In contrast, power, temperature, LOF, and other interactions and main effects did not significantly affect RT values (*p* > 0.05) for either juice.

Based on the statistical indicator values, it is evident that RT can be reliably predicted, with the regression coefficients for RCM and QM detailed in Table S3. The 3D surface plots generated using RSM (Figure S3) demonstrate that RT decreased as MFR increased, since higher MFR leads to an increased flow rate and consequently a reduced RT (Al‐Hilphy et al. 2021). For instance, increasing the MFR from 0.0016 to 0.0079 kg/s at a power input of 1.72 kW and a temperature of 40°C reduced RT from 1.39 and 1.40 min to 0.285 and 0.284 min for OJ and DBLJ, respectively. On the other hand, power and temperature did not show a noticeable effect on RT values. The optimization results presented in Table 2 revealed that RT values of CTS‐OJ and CTS‐DBLJ were significantly higher (*p* < 0.05) than TP by 0.30 and 0.24 min, respectively, which is mainly attributed to the dominant influence of MFR on RT.

#### 3.1.4 Reynolds Number

The Re is an important dimensionless number in fluid mechanics, defined as the ratio of inertial forces to viscous forces. It is a dimensionless quantity that can be used to characterize the flow properties of fluids within pipes. The results presented in Table 1 indicate that the Re values for OJ and DBLJ ranged from 621.41 to 5911.49 and 569.33 to 5351.83, respectively. According to these results, the flow type inside the pipes varied between laminar, transitional, and the onset of turbulence. Singh et al. (2025) stated that when Re < 2100, the flow is laminar; when 2100 < Re < 4000, the flow is transitional; and when Re > 4000, the flow becomes turbulent. The results also revealed that the highest and lowest Re values occurred at MFR of 0.0142 and 0.0016 kg/s, respectively. Tables S2 and S1 showed that the Linear Model (LM) for OJ, DBLJ, MFR, and temperature had a significant effect (*p* < 0.05), while the effects of power and LOF were non‐significant (*p* > 0.05) on the Re values of the treated juices. The statistical indicators, with *R* ^2^ = (0.99, 0.99), Adjusted *R* ^2^ = (0.99, 0.99), and Predicted *R* ^2^ = (0.99, 0.99) for OJ and DBLJ, respectively, indicated that Re values could be predicted. The regression coefficients for LM are provided in Table S3. As shown in Figure S4, Re increased with MFR, as an increase in the latter corresponds to a higher discharge rate, thereby increasing Re (Al‐Hilphy et al. 2021; Alsaedi et al. 2023). For example, increasing the MFR from 0.0016 to 0.0079 kg/s at power 1.676 kW and a temperature of 30°C resulted in a rise in the Re values from 736.66 and 640.38 to 5905.15 and 5303 at the same power and temperature for OJ and DBLJ, respectively. Optimization results in Table 2 showed no significant differences (*p* > 0.05) between the experimental and predicted Re values. Additionally, the flow was classified as laminar according to Singh et al. (2025).

#### 3.1.5 Productivity (L/h)

The CCD matrix presented in Table 1 revealed that the Pr values for OJ and DBLJ ranged between 30.21–56.25 L/h and 28.55–55.58 L/h, respectively. The results indicated that the highest and lowest productivity values were observed at runs 1 and 20 for both OJ and DBLJ. As shown in Table S1, the variables QuM, MFR, and temperature, as well as the interaction between MFR and temperature, and the quadratic interactions of power with MFR, power with temperature, and power with the squared MFR, had a statistically significant effect (*p* < 0.05) on Pr values for OJ. In contrast, LOF and the remaining factors and their interactions did not exhibit any significant influence (*p* > 0.05). Regarding DBLJ, the results presented in Table S2 demonstrated that QM, MFR, temperature, the interaction between MFR and temperature, the quadratic terms of MFR and temperature, and LOF had significant effects (*p* < 0.05) on Pr, whereas power and the remaining variables and interactions did not show any significant effect (*p* > 0.05). Based on the statistical indicators for OJ and DBLJ, such as *R* ^2^ = (0.99, 0.97), Adjusted *R* ^2^ = (0.99, 0.99), and Predicted *R* ^2^ = (0.99, 0.98), respectively, the model was found to be highly predictive for OJ productivity. However, prediction was less reliable for DBLJ due to the significance of LOF. The regression coefficients for QuM and QM are presented in Table S3. As illustrated in Figure S5, an increase in MFR coupled with a decrease in temperature led to higher Pr values. This trend can be attributed to the influence of these parameters on the time required to reach the treatment temperature within the water bath, as well as the residence time of the juice in the CTS. Specifically, higher treatment temperatures necessitated longer heating times, which adversely affected Pr. For example, increasing the MFR from 0.0016 to 0.0142 kg/s at a fixed power level of 1.764 kW and treatment temperature of 50°C resulted in a rise in Pr from 30.21 and 28.41 L/h to 35.78 and 33.19 L/h for OJ and DBLJ, respectively. Improvement results shown in Table 2 indicated that the Pr values for CTS‐treated OJ and DBLJ were significantly higher (*p* < 0.05) than those obtained via TP, with increases of 19.32 and 18.56 L/h, respectively. This enhancement is likely due to the reduced time required for the juices to reach the target treatment temperatures of 45.13°C and 46.68°C for CTS‐OJ and CTS‐DBLJ, respectively, in comparison to TP, which required a considerably longer time to achieve 90°C.

#### 3.1.6 Continuous‐Use Simulation for Temperature and Acoustic Intensity of CTS

Figure S6 illustrates ultrasonic pasteurizer temperature and acoustic intensity simulation for two variables over an 8 h time span. The temperature starts around 25°C at time 0. It rises gradually to a peak of approximately 35°C near 4 h. Then it decreases symmetrically back to 25°C by 8 h. This depicts the thermal profile inside the ultrasonic pasteurizer during continuous operation, simulating heating and cooling phases. The acoustic intensity starts at its maximum value of 1 at time 0. It decreases exponentially to nearly 0 by 5 h and remains negligible onward. This simulates the decay of ultrasonic power or cavitation intensity over time, possibly due to attenuation, transducer heating, or energy loss. At the beginning, high acoustic intensity generates heat via cavitation, raising the temperature. As acoustic intensity declines, heat input reduces, causing temperature to peak and then fall as cooling dominates. The curve shows the coupling between thermal behavior and ultrasound power over continuous use. The temperature transient mimics pasteurization heating, critical for microbial inactivation, followed by cooling or steady state. The exponential acoustic intensity decay models degradation or energy dissipation of ultrasound during prolonged operation.

### 3.2 Effect of CTS on Physicochemical Properties

#### 3.2.1 The pH Vale

The CCD matrix in Table S4, the ANOVA results in Tables S5 and S6, as well as the 3D RSM‐drawn Figure S7 showed slight differences in pH values across all treatments, with pH values ranging from 4.32–4.34 and 3.35–3.36 for OJ and DBLJ, respectively. These results were within the normal range for OJ indicated by Spira et al. (2018) of 4.06, while for DBLJ, it was higher than what Aboud et al. (2020) found at 2.91 for the fresh juice sample. Accordingly, it is difficult to use LM to predict pH values due to weak statistical criteria, as the regression coefficient values are shown in Table S7. These results are consistent with those of Kalsi, Singh, Alam, and Bhatia (2023), who reported that the pH level of guava juice did not significantly change (*p* > 0.05) after treatment with TS. These results can be explained by the fact that TS cannot modify the structure associated with physical properties at the microscopic level (Kalsi, Singh, and Alam 2023). The optimization results in Table 2 showed no significant differences (*p* > 0.05) between CTS‐OJ, CTS‐DBLJ, and the fresh juice sample. In contrast, the TP treatment recorded a significant decrease (*p* < 0.05) in pH. These results were consistent with the findings of Çöl et al. (2023), who recorded a significant decrease (*p* < 0.05) in pH after treating grape juice with TP, while there were no significant differences (*p* > 0.05) for the sample treated with TS.

#### 3.2.2 Titratable Acidity (%)

The results of the CCD matrix are shown in Table S4, ANOVA in Tables S5 and S6 and 3D Figure S8 plotted by RSM as well as the optimization results are shown in Table 2. The TA% values were not significantly affected (*p* > 0.05) after CTS treatment, while the differences were significant (*p* < 0.05) for TP treatment. The TA values for the CCD array ranged between 0.774–0.806 and 0.96–0.998% for OJ and DBLJ, respectively. These results were close to the normal range for OJ recorded by Amaro and Tadini (2021) at 0.746% and Abdulla et al. (2021) 0.875%, while for DBLJ it was higher than what was reached by Aboud et al. (2020) at 0.78% for the fresh juice sample. According to the regression equation values given in Table S7 and the statistical parameters given in Tables S5 and S6, it is difficult to use LM and QuM models to predict TA% values. These results are in agreement with Sahu, Kumar, Minz, et al. (2023) on Nagpur Mandarin Juice and Kalsi, Singh, Alam, and Bhatia (2023) on Guava Juice, where the authors indicated that TA was not significantly affected (*p* > 0.05) after treating juices with TS. This indicates that TS maintained the basic composition of organic acids.

#### 3.2.3 Color Properties (L*, a*, b*, and ΔE)

Table S4 shows that the L* values ranged between 73.65–74.33 for OJ and 72.81–73.52 for DBLJ, respectively. The results also revealed that the highest and lowest L* values were observed at Runs 3 and 9 for both OJ and DBLJ. The ANOVA results in Tables S5 and S6 indicated that LM, MFR, and temperature had significant effects (*p* < 0.05) on L* values, while LOF and the remaining factors and interactions did not show significant influence (*p* > 0.05). Statistical indicators suggest that the LM can be used to predict L* values, as evidenced by the regression equations provided in Table S7. These findings are in agreement with Han et al. (2024), who reported a significant decrease (*p* < 0.05) in L* values of lily juice with increasing US power levels. The 3D response surface plot in Figure S9 illustrates that increasing power, decreasing MFR, and increasing temperature were associated with reductions in L* values. These results align with the findings of Oladunjoye and Awani‐Aguma (2023), who recorded a significant decrease (*p* < 0.05) in the L* values of mango juice with increasing applied power, temperature, and treatment time, where L* values ranged from 39.23–42.04 compared to 43.24 in untreated samples. The reduction was attributed to isomerization reactions caused by cavitation, which are highly dependent on processing conditions. Energy input, treatment duration, and temperature are key factors influencing the color of processed juices (Abdulstar et al. 2023). According to the results presented in Table S4, the a* values for OJ and DBLJ ranged from −4.14 to −4.68 and from 16.02 to 16.58, respectively. Moreover, as shown in Tables S5 and S6, the LM for both OJ and DBLJ indicated that power input, MFR, and temperature had statistically significant effects on the a* values (*p* < 0.05), whereas the LOF was not significant (*p* > 0.05). Based on statistical parameters such as *R* ^2^ (0.97 and 0.95), adjusted *R* ^2^ (0.97, 0.94), and predicted *R* ^2^ (0.96, 0.94) for OJ and DBLJ, respectively, the LM was deemed reliable for predicting a* values. The regression coefficients for the models are provided in Table S7. These results are consistent with those of Qiu et al. (2024), who reported a significant decrease (*p* < 0.05) in the a* values of blackcurrant juice treated with TS. As shown in the 3D surface plots (Figure S10), a* values decreased in response to increasing power, decreasing MFR, and rising temperature. For instance, reducing MFR from 0.0142 to 0.0016 kg/s at 1.72 kW and 40°C led to a decrease in a* values from −4.39 to −4.48 for OJ and from 16.35 to 16.21 for DBLJ under the same power and MFR conditions. These findings align with the results of Kalsi, Singh, Alam, and Bhatia (2023), who observed a significant (*p* < 0.05) reduction in a* values in guava juice with increased treatment time and temperature. Cavitation phenomena may have led to the formation of additional pigmented compounds, as suggested by Oladunjoye et al. (2021), or could be associated with chemical reactions, enhanced diffusion rates, pigment polymerization, or pigment degradation induced by TS, as noted by Xu et al. (2023). According to the data presented in Table S4, b* values ranged from 70.83 to 71.7 and from 39.82 to 40.54 for OJ and DBLJ, respectively. The highest and lowest values for both OJ and DBLJ were recorded at Run 3 and Run 9. As shown in Tables S5 and S6, LM parameters, including power, MFR, and temperature, had a statistically significant effect (*p* < 0.05) on b* values, whereas the LOF was not significant (*p* > 0.05). These findings are consistent with those of Liao et al. (2020), who reported a significant decrease (p < 0.05) in b* values of pitaya juice with increasing applied energy, temperature, and treatment time. The LM fit indices for the experimental data were high, indicating the possibility of using LM to predict b* values, as the values of the regression equations are shown in Table S7. The 3D RSM plots (Figure S11) also revealed a simultaneous decrease in b* values with increasing power, temperature, and decreasing MFR. For instance, increasing power from 1.676 to 1.764 kW at a fixed MFR of 0.0142 kg/s and temperature of 50°C resulted in a decline in b* values from 71.56 to 70.88 in OJ and from 40.4 to 39.86 in DBLJ. Regarding total color difference (ΔE), the CCD matrix shown in Table S4 revealed that ΔE values for OJ and DBLJ ranged from 0.03 to 1.26 and from 0.041 to 1.197, respectively. Based on previous studies, ΔE is influenced by various factors such as natural pigment concentration in juices, the type and conditions of the treatment, changes in other color parameters (a* and b*), as well as the measurement methods employed. As illustrated in Tables S5 and S6, LM parameters including power, MFR, and temperature significantly affected (*p* < 0.05) ΔE values in both OJ and DBLJ, while LOF did not show any significant effect (*p* > 0.05). These results are consistent with those of Liao et al. (2020), who reported a significant increase (p < 0.05) in ΔE in pitaya juice with increasing energy input, temperature, and treatment duration. Similar observations were made by Menelli et al. (2021) in strawberry juice and Cao et al. (2019) in cranberry juice. Based on the regression equations in Table S7 and statistical indicators in Tables S5 and S6, LM can effectively predict ΔE values in treated juices. The 3D RSM plot (Figure S12) demonstrated a synchronous increase in ΔE with increasing power and temperature and decreasing MFR. For example, decreasing the MFR from 0.0142 to 0.0016 kg/s at a power of 1.764 kW and a temperature of 50°C led to an increase in ΔE from 1.19 and 1.13 to 1.3 and 1.24 for OJ and DBLJ, respectively. These findings align with Wang et al. (2020), who reported a significant increase (*p* < 0.05) in ΔE values with extended treatment times (4–16 min) at 400 W and 25 kHz. According to Cserhalmi et al. (2006), ΔE values between fresh and treated juices can be classified as follows: imperceptible (0 < ΔE < 0.5), slightly noticeable (0.5 < ΔE < 1.5), noticeable (1.5 < ΔE < 3), clearly visible (3 < ΔE < 6), and highly noticeable (6 < ΔE < 12). All CTS‐treated juice samples exhibited color changes ranging from imperceptible to slightly noticeable. The optimization results in Table 2 showed that CTS maintained the color parameters (L*, a*, b*, and ΔE) better than TP, with a slightly significant difference (*p* < 0.05) from fresh juice. These results were consistent with Li et al. (2019) on Chinese cranberry juice, Ozyurt et al. (2019) on apple juice, Xu et al. (2023) on strawberry juice, and Hoque et al. (2024) on pineapple juice.

### 3.3 Estimation of Microbial Parameters

#### 3.3.1 Activity of Pectin Methyl Esterase Enzyme and Its Residual Activity

**FIGURE 4:**
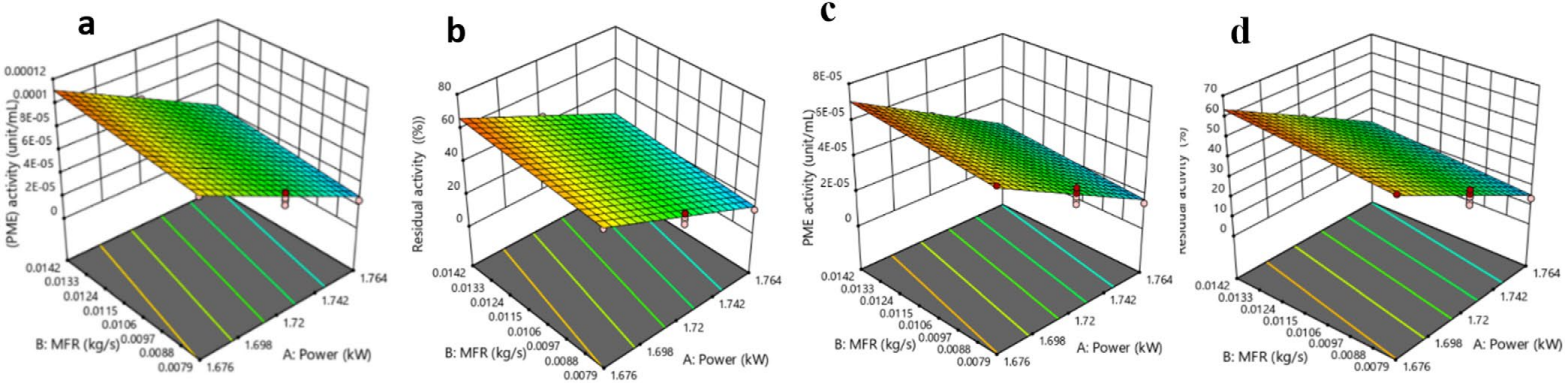
Response surface plots showing the effect of power and MFR on the PME (unit/mL) and RA (%). (a, b) OJ. (c, d) DBLJ.

Table 3 presents the CCD matrix illustrating the effects of power, MFR, and temperature on the activity of pectin methylesterase (PME, unit/mL) and its residual activity percentage (RA%) in OJ and DBLJ following treatment with the CTS. The results showed that PME activity and RA% ranged from 0.0000097 to 0.00012 unit/mL and 5.83% to 71.17% for OJ, respectively. In contrast, DBLJ values ranged from 0.0000053 to 0.000074 unit/mL and 4.73% to 66.67%, respectively. The lowest and highest values were observed at experimental runs 9 and 3, respectively. According to the ANOVA results detailed in Tables S8 and S9, the LM for both OJ and DBLJ showed significant effects (*p* < 0.05) for the factors power, MFR, and temperature. However, the LOF was not significant (*p* > 0.05), indicating a good model fit for the data. Based on the statistical parameters shown in the Supporting Information S1, the LM could be effectively used to predict PME activity and RA%, and the corresponding regression equations are provided in Table S10. As shown in the 3D RSM plots (Figure 4), PME activity and RA% decreased simultaneously with an increase in power and a decrease in MFR for both OJ and DBLJ. For example, increasing power from 1.676 to 1.764 kW at a constant MFR of 0.0079 kg/s and temperature of 40°C reduced PME activity and RA% in OJ and DBLJ from 0.000098 and 0.000065 unit/mL and 57.92% and 55.69%, respectively, to 0.000024 and 0.000016 unit/mL and 17.11% and 15.45%, respectively. These findings are consistent with those of Tewari et al. (2024), who observed a significant (*p* < 0.05) reduction in RA with increased applied energy levels. In their study, RA ranged from 4.50% to 91.35% in amla pulp treated with energy levels ranging from 180 to 717 W/cm^2^ for durations between 4 and 16 min. Evaluated the effect of continuous thermosonication treatment (Probe) on the activity of some enzymes in skimmed milk, namely alkaline phosphatase, glutamyltranspeptidase, and lactoperoxidase. The results showed that treatment at (amplitude 120 μm, frequency 20 kHz, power 150 W, temperature 75.5°C, treatment time 102.3 s) resulted in a significant decrease in the residual activity by 0%, 0%, and 47.2%, respectively, compared to the conventional treatment (70 m for 102.3 s), which amounted to 0%, 8.5%, and 62.9%, respectively. The mechanism of enzyme inhibition during TS is due to a combination of physical, chemical, and thermal effects. As ultrasound waves pass through a liquid medium, the subsequent compression and rarefaction cycles generate physical forces that lead to the formation of cavitation bubbles. These bubbles, as they move, generate strong eddy currents in the medium, transmitting constant shear forces. As a result, each bubble formed is significantly affected by the shock generated by the neighboring bubble, rendering it unstable and leading to its collapse. These forces are called the sono‐physical effect (Yildiz and Yıldız 2025). These forces expose protein secondary structures and help expose internally buried hydrophobic amino acids (Yu et al. 2014). This results in increased hydrophobicity of the surface of the ultrasound‐treated enzyme proteins, thereby increasing the interaction between protein molecules through disulfide bonds and hydrophobic interactions. These interactions facilitate protein aggregations, which contribute to masking the substrate binding site of the enzyme, thereby inactivating it (Rathnakumar et al. 2023). For example, the structural characterization of fungal PPO treated with ultrasound showed partial degradation of the enzyme protein (Baltacıoğlu et al. 2017). The sonic thermodynamic effects are due to eddy currents and the energy released upon bubble collapse, resulting in localized hot spots (> 5000 K), ultimately leading to degradation of the enzyme protein and loss of its activity due to intense energy transfer. Meanwhile, the chemical effects represented by the formation of free radicals such as O_2_•, OH^•^, and HOO^•^ resulting from the dissociation of water molecules cause the oxidation of some amino acids such as tyrosine, tryptophan, cystine, and histidine, ultimately leading to an increased enzyme inhibition rate (Manzoor et al. 2023; Tomadoni et al. 2016). For example, the oxidation of amino acids in enzymes after ultrasonic treatment was efficiently studied by assessing the fluorescence intensity of the enzymes. It was reported that the fluorescence intensity contributed by aromatic amino acids (tyrosine, tryptophan) decreased after ultrasonication, indicating the potential for amino acid oxidation (Tsikrika et al. 2022). In addition to the phenomenon of amino acid oxidation, the produced radicals also cause significant changes in conformation and aggregation. Active free radicals can collide with disulfide bonds in proteins and convert them to a thiol moiety at the terminal, altering the conformation of the enzyme protein (Rathnakumar et al. 2023). Since enzyme activity is primarily dependent on its conformation, any change in the conformation inactivates the enzyme. In addition, the secondary structure of proteins is also affected by hydrogen bond cleavage caused by ultrasound (Rathnakumar et al. 2023). Optimization results in Table 2 showed no significant differences (*p* > 0.05) between experimental and predicted values of PME activity and RA%, confirming the reliability of the predictive models. Moreover, both CTS‐OJ and CTS‐DBLJ treatments exhibited high efficiency and performance comparable to TP in reducing RA%. For instance, RA% in OJ samples decreased to 7.48% and 6.98% after CTS‐OJ and TP treatments, respectively, while RA% for DBLJ samples declined to 6.42% and 5.95% following CTS‐DBLJ and TP treatments, respectively. These results are in line with those reported by Kalsi, Singh, Alam, and Bhatia (2023), who successfully reduced PME residual activity to 6.07% in guava juice treated with TS at 200 W and 60°C for 10 min, compared to 5.91% under conventional pasteurization (90°C for 60 s). However, these results were more effective than those observed by Sahu, Kumar, and Kiran (2023) in Nagpur mandarin juice, where RA% was 27.44% under TS treatment (40 kHz, 60°C, 15 min) compared to 30.6% with conventional treatment. The authors attributed this to the synergistic effects of ultrasound and heat. Koshani et al. (2015) noted that the synergistic effect is less efficient at temperatures above 70°C, which aligns with findings by Wu et al. (2008), who reported that TS is most effective at moderate temperatures. This could be due to the increased vapor pressure of water at elevated temperatures, which results in less violent bubble collapse (Sala et al. 1995). Finally, it is important to note that variations in the outcomes of previous studies can be attributed to several factors, including fruit type, maturity stage, treatment conditions, enzyme activity, and analytical methods.

**TABLE 3:** CCD matrix for the effect of power, MFR, and temperature on the enzymatic and microbial properties of OJ and DBLJ treated with CTS.

RUN	Independent variables						Dependent variables				
	OJ						DBLJ				
	P	MFR	T	PME	RA	TBC	*D*‐value	PME	RA	TBC	*D*‐value
1	1.72	0.0142	40	0.000076	45.45	2.52	0.4364	0.000047	42.59	2.73	0.3294
2	1.72	0.0079	50	0.00005	29.81	2.32	0.5045	0.00003	27.27	2.54	0.4264
3	1.676	0.0142	30	0.00012	71.17	2.68	0.7852	0.000074	66.67	2.93	0.569
4	1.72	0.0079	40	0.000065	38.83	2.41	0.601	0.00004	36	2.66	0.5184
5	1.764	0.0142	30	0.000062	36.99	2.38	0.3142	0.000039	35.28	2.64	0.2779
6	1.676	0.0016	50	0.000077	46.15	2.43	3.0985	0.000049	44.47	2.75	3.0311
7	1.72	0.0079	30	0.000074	44.58	2.49	0.7239	0.000049	43.77	2.72	0.5727
8	1.764	0.0016	30	0.000015	8.88	1.85	1.3535	0.0000087	7.826	2.04	1.198
9	1.764	0.0016	50	0.0000097	5.83	ND	0.4845	0.0000053	4.73	ND	0.4379
10	1.764	0.0142	50	0.000031	18.55	1.48	0.1122	0.000018	16.36	2.3	0.1739
11	1.72	0.0079	40	0.000061	36.88	2.4	0.5885	0.000038	34.62	2.65	0.5101
12	1.72	0.0079	40	0.000066	39.68	2.43	0.6274	0.000036	32.14	2.62	0.4825
13	1.72	0.0079	40	0.000055	32.75	2.36	0.5433	0.000042	37.5	2.67	0.5274
14	1.72	0.0016	40	0.000056	33.33	2.28	2.3245	0.000035	31.62	2.6	2.2989
15	1.676	0.0142	50	0.0001	60	2.6	0.5611	0.000065	58.67	2.88	0.47
16	1.764	0.0079	40	0.000025	14.96	1	0.1503	0.000015	13.59	2.2	0.2819
17	1.72	0.0079	40	0.000065	39.14	2.34	0.523	0.000043	38.25	2.68	0.535
18	1.676	0.0016	30	0.00009	54.55	2.54	4.0997	0.000059	52.94	2.83	3.6928
19	1.72	0.0079	40	0.000059	35.29	2.45	0.6567	0.000034	30.75	2.59	0.4571
20	1.676	0.0079	40	0.000096	57.42	2.58	0.9412	0.000063	56.25	2.85	0.7738

#### 3.3.2 Total Count of Bacteria (Log CFU/mL)

The CCD matrix presented in Table 3 showed that the TBC ranged between 2.68 and undetectable levels and 2.93–ND log CFU/mL for OJ and DBLJ, respectively. The results demonstrated that TBC decreased with increasing power, temperature, and decreasing MFR. The highest TBC values were recorded at *p* = 1.676 kW, MFR = 0.0142 kg/s, and T = 30°C, whereas treatment at power = 1.764 kW, MFR = 0.0016 kg/s, and temperature = 50°C reduced TBC to undetectable levels in both OJ and DBLJ. These findings are consistent with those reported by Qiu et al. (2024), who observed a reduction in TBC with increased treatment time and temperature, achieving complete microbial inactivation in blackcurrant juice treated with TS at 480 W, 40 kHz, and 50°C for 10 min. A study conducted by Deshpande and Walsh (2020) on the effect of continuous system ultrasound on the microbial quality of milk showed that treatment with this technique (60% amplitude, 234.44 power, 72°C temperature, 11.1 s treatment time and 0.76 L/min flow rate) resulted in a significant decrease in the total bacterial count by 4.06 log CFU/mL in milk compared to a decrease of 2.79 for the heat treatment (72°C for 11.1 s). The microbial inactivation mechanisms of TS can be attributed to the synergistic effects between moderate heat and US, where moderate heat weakens the bacterial cell membrane, rendering it more susceptible to US. Meanwhile, cavitation events induce significant pressure variations, resulting in bubble formation. Upon collapse during compression cycles, these bubbles generate localized regions with pressures up to 100 MPa and temperatures ranging from 5000 to 15,000 K, without elevating the bulk temperature of the product. Additionally, sonolysis of water produces free radicals (H+, O2•, OH•, HOO•) that cause oxidative damage to microbial cells (Basumatary et al. 2020). The efficiency of TS in microbial inactivation depends on several parameters such as applied ultrasound power, frequency, temperature, treatment duration, microbial strain, and product pH (Bhutkar et al. 2024). As shown in Table S8 for OJ, a significant effect (*P* < 0.05) was found for QuM, power, MFR, power × MFR, power × temperature, MFR × temperature, power square, the triple interaction, power square ×MFR, power square × temperature, power × MFR square, and power square × MFR square on TBC values. However, temperature, LOF, and other interactions did not show significant effects (*p* > 0.05). These results are consistent with Deshpande and Walsh (2021) who investigated the effect of continuous thermosonication treatment (Probe) on total bacterial count in milk. The results showed that increasing the exposure time to ultrasound from 7.1 to 11.9 s at 90% amplitude and 72°C resulted in a significant improvement in the logarithmic decline from 1.21 to 1.91. Regarding DBLJ, results in Table S9 indicated significant effects (*p* < 0.05) of QuM, power, MFR, power × MFR, power× temperature, MFR× temperature, the triple interaction, power square × temperature, power × MFR square, and power square ×MFR on TBC values, while temperature, LOF, power square, and other interactions did not significantly affect TBC (*p* > 0.05). Based on the statistical indicators, the regression model can be used to predict TBC values, with regression coefficients for QuM presented in Table S10. The 3D surface plots in Figure S13, generated using RSM, reveal that TBC values decreased simultaneously with increasing power and temperature and decreasing MFR. For instance, raising power from 1.72 to 1.764 kW at MFR = 0.0079 kg/s and 40°C reduced TBC from 2.4 and 2.65 to 1 and 2.24 log CFU/mL for OJ and DBLJ, respectively. Optimization results presented in Table 2 confirmed a reduction in TBC from 2.75 and 2.96 log CFU/mL to undetectable levels for OJ and DBLJ, respectively, across all treatments. These outcomes are in agreement with Noorisefat et al. (2025), who reported TBC reductions to undetectable levels in sour cherry juice after TS treatment at 200 W, 40°C for 3 min. The microbial inactivation was attributed to cell membrane rupture and alterations in nucleic acids and protein structures. Similarly, Lv et al. (2024) achieved complete TBC inactivation in *Aronia melanocarpa* juice treated with TS at 700 W, 20–25 kHz, 55°C for 10 min. The enhanced inactivation efficiency of CTS could be attributed to the innovative design of the pasteurization unit, which maximizes surface area exposed to US, particularly at low MFR. This was achieved by using a narrow internal diameter tube (0.2 cm) of appropriate length (40 m), coiled in an elliptical shape and immersed in the ultrasonic bath. This configuration ensures direct and indirect exposure of microbes to US, both from the bath and via water surrounding the tubing.

#### 3.3.3 Weibull Model of TBC

Figure S14 Fitting the Weibull distribution‐based model to the survival curves (upward concavity) of TPC in limon and orange juice treated by TS. The results illustrated that the $\log_{10}\left(\frac{N}{N_{o}}\right)$ was reduced as time increased. These results are in agreement with Fernández et al. (2007) and van Boekel (2002), who stated that the log10 survival fraction decreased with increasing time. There is a good fitting for TPC for limon and orange juice with *R* ^2^ = 0.9997, and 0.9997, respectively. From the Weibull model, the predicted N after pasteurization by TS equals 1.95, and 2.04 CFU/mL, respectively.

#### 3.3.4 D‐Values (Min) for TBC

The CCD matrix presented in Table 3 illustrates that the D‐values for OJ and DBLJ ranged from 0.112 to 4.1 min and 0.174 to 3.693 min, respectively. This is based on the initial loadings of the fresh sample, which amounted to (2.88, 3.21) CFU/mL for OJ and DBLJ, respectively. It was also noted that the lowest and highest D‐values were at (Run 10 and 18). The results further revealed that the D‐value for OJ was higher compared to DBLJ. This difference can be attributed to several factors, primarily pH. Specifically, OJ exhibited a higher pH level than DBLJ, with pH values ranging from 4.32–4.34 for OJ and 3.36–3.36 for DBLJ. Generally, microorganisms demonstrate increased resistance to TS under near‐neutral pH conditions compared to acidic environments. This implies that under near‐neutral conditions, more intense processing is required to achieve the same level of microbial inactivation as under acidic conditions. These findings are consistent with those of Araghi et al. (2024), who reported elevated D‐values for *E. coli* K12 in blueberry juice compared to watermelon juice when both were treated at temperatures ranging from 52°C to 58°C. The authors attributed this to the higher pH (3.39 vs. 4.60) and lower titratable acidity (0.34% vs. 0.24%) of blueberry juice compared to watermelon juice. Additionally, the presence of pectic substances in OJ may provide a protective barrier against US, thereby contributing to the higher D‐values observed relative to DBLJ. Boghossian et al. (2023) indicated that the combination of temperature with US at 35 kHz, 480 W, and 55°C–60°C for 30–55 min exerted a synergistic effect in significantly reducing the D‐values of *L. innocua* in kiwi peel, compared to thermal treatment alone at 55°C and 60°C. It is important to note that the efficacy of TS in microbial inactivation is influenced by several factors, including the food matrix and its physicochemical characteristics, the type of target microorganisms, temperature, treatment duration, ultrasound power, and frequency. The results of ANOVA analysis in Tables S8 and S9 showed that RCM of OJ and DBLJ, power, MFR, MFR square, and various interactions such as power × MFR, MFR × temperature, power square × MFR, power square × temperature, power square ×MFR, power square × temperature, and (interaction of power and MFR square except OJ) had a significant effect (*p* < 0.05), while LOF and the rest of the other factors and interactions had no significant effect (*p* > 0.05) on the D‐values of OJ and DBLJ. According to the values of the statistical indicators, the D‐values can be predicted, as the values of the regression coefficients for RCM are shown in Table S10. The 3D surface plots generated by the RSM and illustrated in Figure S15 clearly demonstrate a decrease in D‐values with increasing power, MFR, and temperature. For instance, increasing the power from 1.676 to 1.764 kW at MFR = 0.0016 kg/s and T = 50.

°C reduced D‐values from 3.1 and 3.04 min to 0.485 and 0.437 min for OJ and DBLJ, respectively. Furthermore, the optimization results in Table 2 revealed that the D‐values for the CTS‐OJ and CTS‐DBLJ treatments were significantly higher (*p* < 0.05) than those for TP. This could be attributed to differences in treatment parameters such as applied power, temperature, and the holding time used to calculate the D‐values.

Finally, the figures (Figures S16 and S18) illustrate the normal distribution plots of the dependent variables for orange juice (OJ) and double blended orange juice (DBLJ), respectively, showing the distribution patterns for variables such as SEC, EE, RT, Re, Pr, pH, TA, L*, a*, b*, PME activity, RA%, TBC, and D‐value. Figures S17 and S19 present the plots of residuals versus predicted values for the same dependent variables in OJ and DBLJ, respectively, which are used to assess model adequacy and identify any systematic patterns or anomalies in the residuals.

#### 3.3.5 Psychrotrophes (Ps), Coliform Bacteria (CB), Yeasts and Molds (Y&M)

Table S11 presents the logarithmic counts of microorganisms under the optimal conditions for juices treated with CTS‐OJ and CTS‐DBLJ, compared to TP and fresh samples. The results demonstrated that populations of Ps, CB, and Y&M declined to undetectable levels across all treatments, indicating a high sterilization efficiency. Similar findings were reported by Lv et al. (2024), who observed the reduction of CB and Y&M to undetectable levels following treatment of *Aronia melanocarpa* juice using TS. These results also align with those of Ma et al. (2022), who reported that Y&M counts dropped to undetectable levels in *Prunus mume* juice after US treatment at 200 W for 15 min. Consistent outcomes were also documented by Abedelmaksoud et al. (2019) for OJ, where complete inactivation of Y&M was achieved using US treatment at 550 W for 8 min. While another study investigated the effect of continuous non‐flow thermosonication (continuously stirring the juice at 500 rpm using a magnetic stirrer) on the inactivation of *E. coli* ATCC 25922 in blackberry juice, the results showed a 5‐log reduction of *E. coli* ATCC 25922 at an acoustic intensity of 1.63 W/mL, 50°C, and a treatment time of 10.45 min (Dinçer and Topuz 2015). Based on the microbiological results, it can be concluded that the combination of ultrasound and mild heat exhibits antimicrobial effects comparable to those of conventional thermal pasteurization, supporting the potential of this technique as a promising alternative for juice pasteurization.

### 3.4 Analysis of Phenolic Compounds of OJ and DBLJ Using RP‐HPLC

Phenolic compounds are present in a variety of fruit juices as secondary metabolites. These substances play an important role as antioxidants, anticancer agents, and antimicrobial agents. RP‐HPLC analysis of OJ and DBLJ samples revealed 11 and 6 compounds, respectively, as shown in Figures S20b–d and S21a–c. Eight and five phenolic compounds were identified, respectively, by comparing their retention times with standard compounds (Figure S20a). These compounds included hydroxybenzoic acids (Gallic acid), hydroxycinnamic acids (p‐coumaric acid and Ferulic acid), flavanones (Naringenin and Hesperidin), flavonols (Rutin), and flavones (Apigenin and Luteolin). Gallic acid was the most concentrated compound in all treated juice samples, with average contents of 41.12 ± 0.99 ppm and 30.51 ± 0.05 ppm for OJ and DBLJ, respectively (Table 4), followed by the rutin compound with an average content of 32.42 ± 0.15 and 27.52 ± 0.07 ppm, respectively, while the rest of the compounds were found in lower quantities. DBLJ recorded the absence of p‐coumaric acid and naringenin in all treatments. The results also demonstrated a significant (*p* < 0.05) increase in the content of gallic acid, p‐coumaric acid, rutin, apigenin, naringenin, ferulic acid, hesperidin, and luteolin after treating the juices with CT‐OJ and CT‐DBLJ, with increases of (26.06%, 28.15%, 25.90%, 40.25%, 56.08%, 29.83%, 12.60%, 21.12%) and (21.46%, 22.75%, 38.78%, 21.53%, 13.66%, 24.54%) for OJ and DBLJ, respectively. In contrast, TP treatment caused a significant (*p* < 0.05) decrease in all phenolic compounds identified in OJ and DBLJ, with reductions of (20.48%, 19.12%, 21.82%, 14.2%, 27.96%, 16.06%, 10.66%, 6.49%) and (18.27%, 16.46%, 14.14%, 18.54%, 11.21%, 9.59%) for OJ and DBLJ, respectively. These results are consistent with several studies that indicated increased retention of phenolic compounds after treatment with US or TS. For example, Alabdali et al. (2020) reported a significant (*p* < 0.05) increase of 49.13% in the content of gallic acid in pomegranate juice after TS treatment, while conventional pasteurization (72°C for 15 s) resulted in a significant decrease of 21.80%. This confirms the findings of Choo et al. (2023), where the researchers noted a 46.18% improvement in rutin content in noni juice after US treatment at 37 kHz for 60 min at temperatures below 30°C, while conventional pasteurization led to a 19.27% decrease in rutin. The increase in the concentration of phenolic compounds in juices treated with CTS can be attributed to the structural disturbance of the cell wall caused by the phenomenon of cavitation, which causes the generation of huge shear forces resulting from the collapse of bubbles, leading to the release of phenolic compounds from inside the cells to the surrounding environment, as well as the possibility of converting compounds from bound forms to free forms (Das et al. 2024). In contrast, the significant decrease in phenolic compounds due to TP is due to the high temperature that enhances the interaction of polyphenols with O_2_ as a result of the hydroxyl groups entering into the structure of polyphenols (Xu et al. 2023).

**TABLE 4:** Effect of optimization conditions on the concentration of phenolic compounds estimated using HPLC compared to TP in the fresh sample.

Polyphenols (ppm)	CTS‐OJ	TP	Fresh	CTS‐DBLJ	TP	Fresh
Gallic acid	41.12 ± 0.99^a^	25.94 ± 1.60^c^	32.62 ± 1.06^b^	30.51 ± 0.05^a^	20.53 ± 0.07^c^	25.12 ± 0.08^b^
p‐coumaric acid	26.81 ± 0.1^a^	16.92 ± 0.04^c^	20.92 ± 0.06^b^	—	—	—
Rutin	32.42 ± 0.15^a^	20.13 ± 0.11^c^	25.75 ± 0.06^b^	27.52 ± 0.07^a^	18.73 ± 0.05^c^	22.42 ± 0.06^b^
Apigenin	20.94 ± 0.11^a^	12.81 ± 0.18^c^	14.93 ± 0.045^b^	18.93 ± 0.04^a^	11.71 ± 0.03^c^	13.64 ± 0.05^b^
Naringenin	19.37 ± 0.03^a^	8.94 ± 0.04^c^	12.41 ± 0.02^b^	—	—	—
Ferulic acid	22.63 ± 0.07^a^	14.63 ± 0.03^c^	17.43 ± 0.05^b^	20.32 ± 0.05^a^	13.62 ± 0.04^c^	16.72 ± 0.08^b^
Hesperidin	11.52 ± 0.01^a^	9.14 ± 0.05^c^	10.23 ± 0.03^b^	11.15 ± 0.06^a^	8.71 ± 0.07^c^	9.81 ± 0.03^b^
Luteolin	14.91 ± 0.08^a^	11.51 ± 0.04^c^	12.31 ± 0.05^b^	10.91 ± 0.06^a^	7.92 ± 0.08^c^	8.76 ± 0.05^b^

*Note:* Different letters in the same row refer to significant differences at level of 0.05.

### 3.5 Sensory Attributes

**FIGURE 5:**
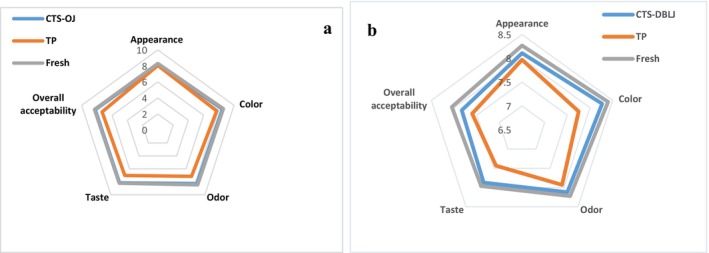
Sensory evaluation of juices treated with CT‐OJ, DBLJ compared to TP and fresh sample: (a) OJ, (b) DBLJ.

Sensory evaluation plays a crucial role in determining the quality and acceptance of products, such as juices and beverages (Song et al. 2022). As shown in Figure 5a, the sensory evaluation results of CTS‐OJ compared to TP and the fresh sample are presented. The results showed no significant differences (*p* > 0.05) between the CTS‐OJ treatment and the fresh sample for all sensory attributes. For instance, the average appearance, color, aroma, taste, and overall acceptability scores for CTS‐OJ were 8.13 ± 0.44, 8.47 ± 0.23, 8.30 ± 0.29, 8.14 ± 0.41, and 8.24 ± 0.37, respectively, while the fresh sample had scores of 8.31 ± 0.31, 8.61 ± 0.23, 8.46 ± 0.18, 8.21 ± 0.28, and 8.32 ± 0.27, respectively. In fact, evaluators could not distinguish between the CTS‐OJ treatment and the fresh sample, providing clear evidence that the applied US energy level and moderate temperatures did not adversely affect the sensory properties of the OJ. These findings align with those of Kalsi, Singh, Alam, and Bhatia (2023) on guava juice and Deli et al. (2022) on cashew apple juice, where they reported that juices treated with TS exhibited sensory properties that were more similar to untreated (fresh) juices. This supports the effectiveness of TS in maintaining sensory attributes through its minimal impact on the molecular structure of flavor compounds. While another study investigated the effect of continuous thermosonication on batches on the sensory properties of processed pumpkin juice at (150 W, 37 kHz frequency and 0.029 L/min flow rate), the results showed a slight significant decrease compared to the untreated samples in some sensory properties such as aroma, flavor, and sweetness, while other qualities such as odor, sourness, color appearance and general acceptance did not show any significant differences compared to the untreated sample (Demir and Kılınç 2019). In contrast, the TP treatment in this study caused a significant decrease (*p* < 0.05) in all sensory attributes except for appearance, compared to the fresh sample and CTS‐OJ. The average scores for appearance, color, aroma, taste, and overall acceptability were 8.07 ± 0.40, 7.73 ± 0.35, 7.15 ± 0.42, 7.01 ± 0.56, and 7.32 ± 0.24, respectively. These results are consistent with Zhang et al. (2024) on lettuce juice, where significant decreases (*p* < 0.05) were observed in all sensory attributes after conventional pasteurization treatment. The reduction can be attributed to the pasteurization heat, which caused changes in taste due to the degradation of flavor compounds and the production of undesirable flavors, as well as color changes due to non‐enzymatic browning and the loss of natural pigments (Anese et al. 2002; Lopes et al. 2016). These results also align with the color analysis results discussed in Section 3.2.3. Regarding DBLJ, the sensory evaluation results shown in Figure 5b revealed that the average scores for appearance, color, aroma, taste, and overall acceptability for the CTS‐DBLJ treatment, TP, and the fresh sample were (8.11 ± 0.32, 8.27 ± 0.42, 8.12 ± 0.43, 7.87 ± 0.35, and 7.83 ± 0.24), (7.97 ± 0.35, 7.75 ± 0.50, 7.93 ± 0.46, 7.43 ± 0.53, and 7.60 ± 0.34), and (8.27 ± 0.56, 8.40 ± 0.34, 8.22 ± 0.35, 7.96 ± 0.28, and 8.05 ± 0.30), respectively. The results indicated that the CTS‐DBLJ treatment did not differ significantly from the fresh sample. These findings are consistent with Yıkmış et al. (2025) on black carrot juice. In contrast, the TP treatment recorded a significant decrease (*p* < 0.05) in all sensory attributes compared to the CTS‐DBLJ and fresh samples, except for appearance and aroma. Aboud et al. (2020) reported a significant decrease (p < 0.05) in the taste attribute after DBLJ treatment with TP. Based on the previous results, it can be concluded that the impact of sensory attributes in treated juices depends on several interrelated factors, including the type of juice, treatment conditions, and treatment type. This impact can be either positive or negative, depending on these factors. For example, moderate conditions help preserve or even intensify aromatic compounds due to cavitation and its role in reducing oxygen content in juices (Yıkmış et al. 2019). Conversely, harsh treatment conditions may lead to the degradation of aromatic compounds due to the physical stresses induced by bubble collapse during cavitation. This was documented by Qiu et al. (2024), who observed a decline in sensory indicators for black currant juice treated with TS after raising the temperature to 60°C at an energy level of 480 W and 40 kHz, with a 40‐min hold time.

## 4 Conclusion

The CTS, as a sustainable processing technology, successfully fulfilled its intended objectives. It significantly reduced SEC and enhanced the process rate Pr. A strong correlation was observed between the predicted and experimental values, confirming the robustness of the applied model. Optimization of the CTS parameters using RSM further improved system performance and juice quality, emphasizing the critical role of mathematical optimization in process engineering. Microbiological evaluations confirmed that CTS was almost as effective as TP in reducing microbial and enzymatic activity. Notably, CTS treatment enhanced the retention of bioactive compounds, whereas TP caused significant degradation of phenolics, likely due to thermal sensitivity. Sensory analysis also revealed that CTS maintained the sensory attributes of the juices, while TP led to a significant decline (*p* < 0.05) in most sensory parameters. However, there is an urgent need for further structural and spectroscopic studies to explore the synergistic effects of the CTS system on a wide range of food enzymes. We also recommend expanding the study to include some potent pathogens and studying the shelf life of treated juices during storage. We also recommend validating the system in different juice matrices, expanding the scope of the study to include some potent pathogens, and studying the shelf life of processed juices during storage. We also believe it is important to conduct a comprehensive analysis of the costs associated with implementing this system in the production process. Furthermore, it would be beneficial to expand the scope of the sensory evaluation panel to include a larger number of individuals, which would enhance the reliability of the results and contribute to a more accurate assessment of the quality of processed juices.

## Author Contributions

**Alaa R. Abdulstar:** methodology, formal analysis, data curation, investigation, writing – original draft, writing – review and editing. **Asaad R. Al‐HiIphy:** methodology, formal analysis, data curation, investigation, writing – original draft, writing – review and editing. **Ammar B. Altemimi:** methodology, formal analysis, data curation, investigation, writing – original draft, writing – review and editing. **Rawaa H. Tlay:** methodology, software, formal analysis, writing – original draft, writing – review and editing. **Tarek Gamal Abedelmaksoud:** methodology, software, formal analysis, writing – original draft, writing – review and editing.

## Disclosure

Author statement: All authors have read and approved the submitted version of the manuscript.

## Conflicts of Interest

The authors declare no conflicts of interest.

## Supporting information


**Data S1:** fsn371171‐sup‐0001‐Supinfo.zip.

## Data Availability

Data will be made available on reasonable request.
