# Supplementary material for: Optimized Continuous Thermosonication for Sustainable Pasteurization of Orange and Dried Black Lime Juices: Techno‐Functional, Physicochemical, and Microbial Assessment
Source: Food Sci Nutr. 2025 Nov 9;13(11):e71171. doi: 10.1002/fsn3.71171 (PMC12597978; doi:10.1002/fsn3.71171)
Supplement: Supplementary file 1 — Data S1: fsn371171‐sup‐0001‐Supinfo.zip. [file FSN3-13-e71171-s001.zip › fsn371171-sup-0001-Figures.docx]

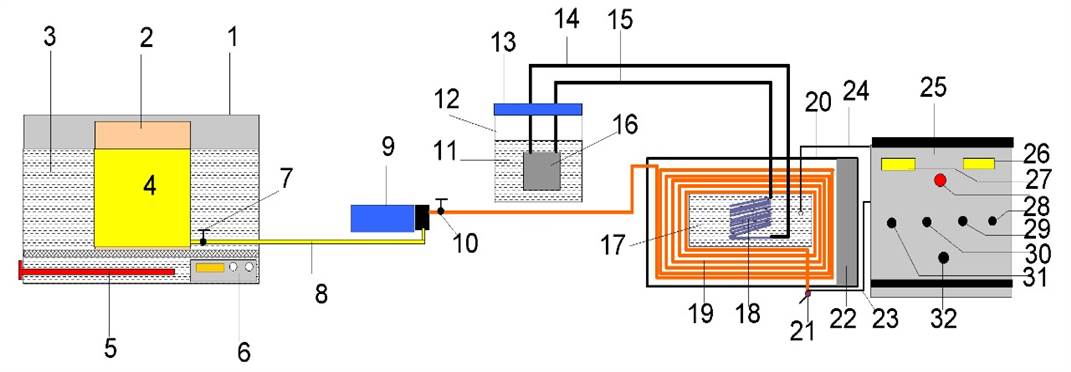


1. Water bath, 2. Juice container cover, 3. Water bath basin water, 4. Juice container, 5. Heating element, 6. Control panel, 7. Valve, 8. Plastic tube, 9. Pump, 10. MFR control valve, 11. Heat exchange unit water, 12. Heat exchange unit pot, 13. Pot cover, 14. 15. Two plastic tubes, 16. Submersible pump, 17. US basin water, 18. Heat exchanger, 19. Plastic tube (oval coiled 40 meters long), 20. US device, 21. Juice outlet valve, 22. US device control panel, 23. Thermocouple connected to juice outlet pipe, 24. Thermocouple connected to ultrasonic basin, 25. Power supply unit, 26. Digital controller gauge for adjusting water temperature inside ultrasonic basin, 27. Digital controller To measure the temperature of the outgoing juice, 28. Digital meter for measuring voltage and current, 29. US device operation switch, 30. Pump operation switch, 31, 32. Two switches for operating the two digital temperature gauges. 33. Main control switch

**FIG. S1 |** Schematic diagram of the locally designed CTS device

**
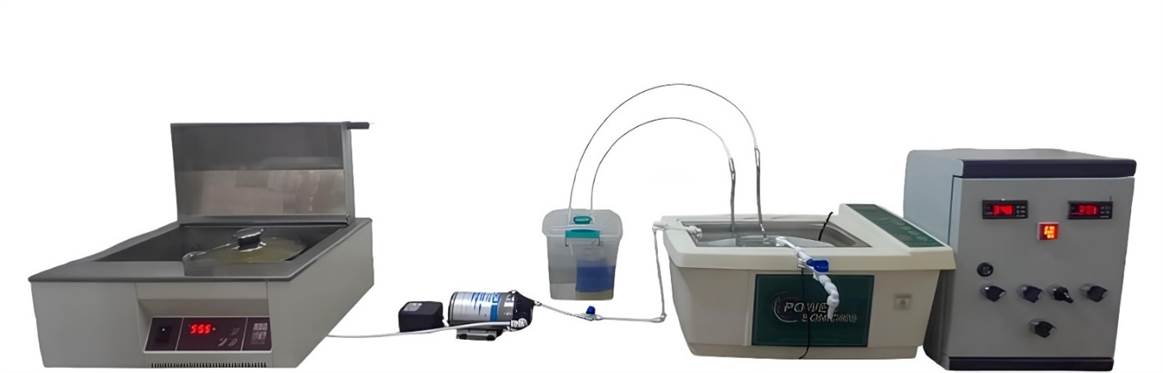
**

**Fig. S2 |** The locally designed CTS device.


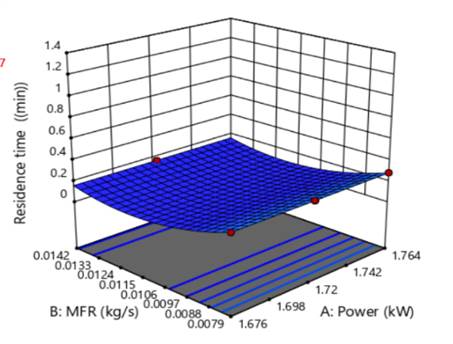

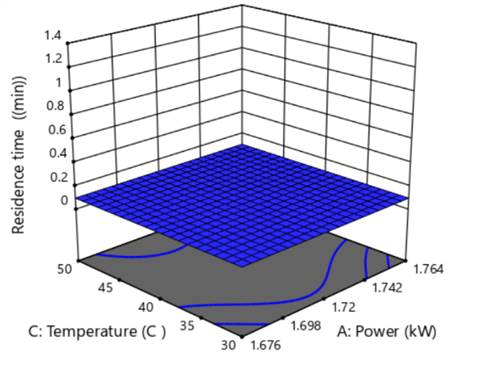

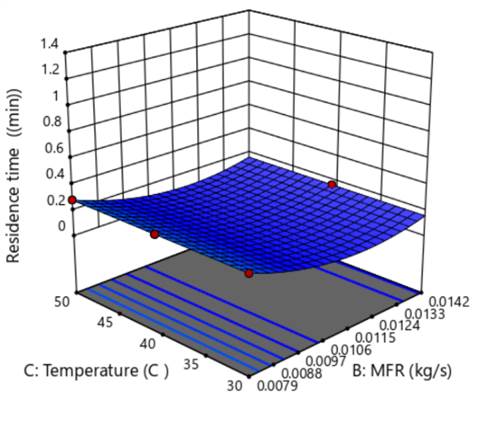


**b**

**a**

**c**

**(˚C)**


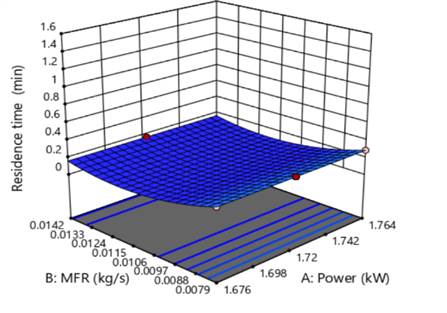

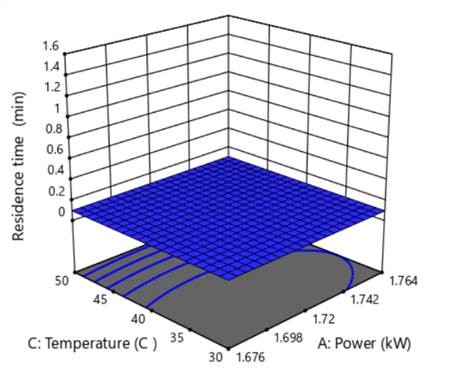

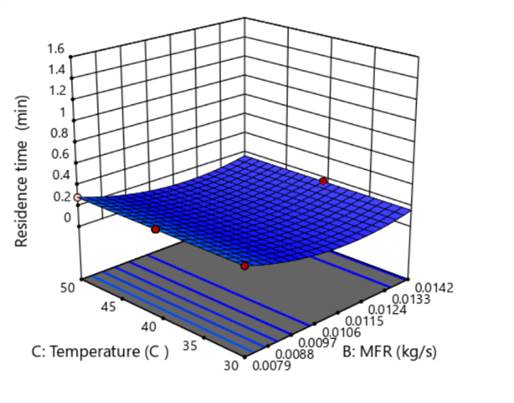


**e**

**d**

**f**

**(˚C)**

C-Temperature (**˚C)**

**˚**

**Fig. S 3 |** Response surface plots showing the effect of independent variables on RT (min), (a,b and c): interactions between independent factors for OJ, (d, e and f): interactions between independent factors for DBLJ.


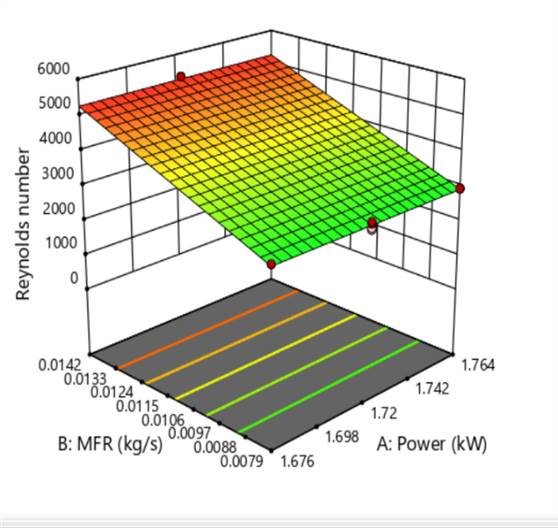

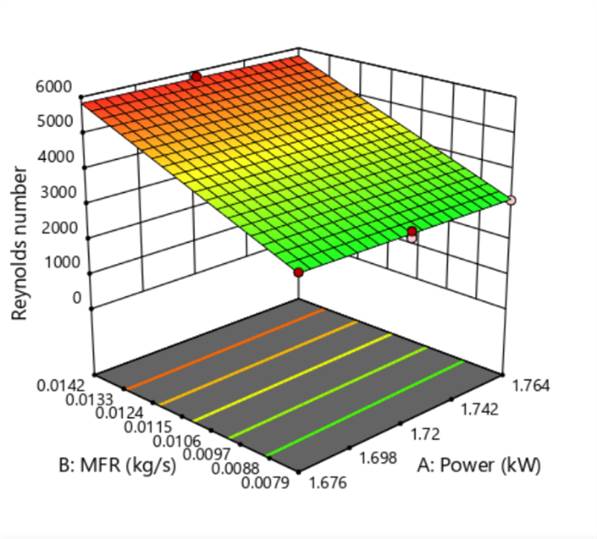


**b**

**a**

**Fig. S 4 |** Response surface plots shows the effect of power and MFR on the Re, a: OJ, b: DBLJ.


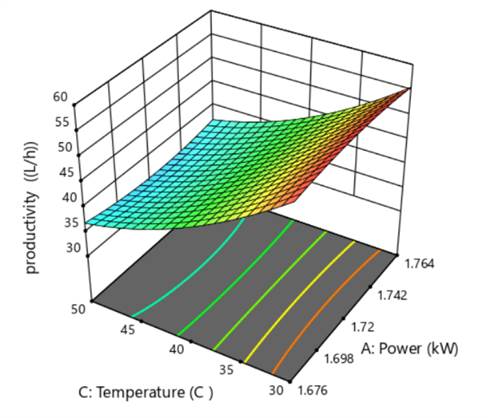


**c**

d

**(˚C)**


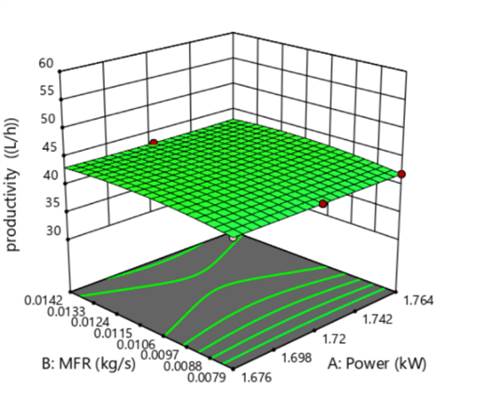


**a**


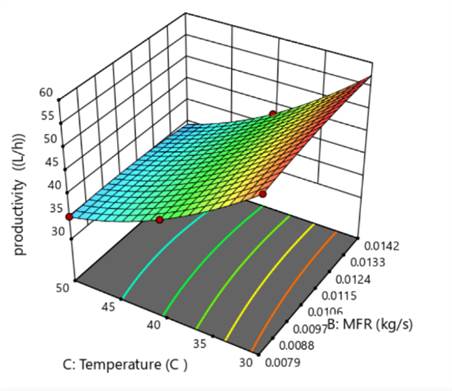


b

d

**(˚C)**


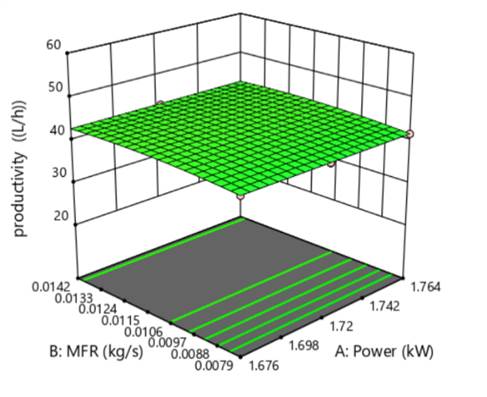


**d**


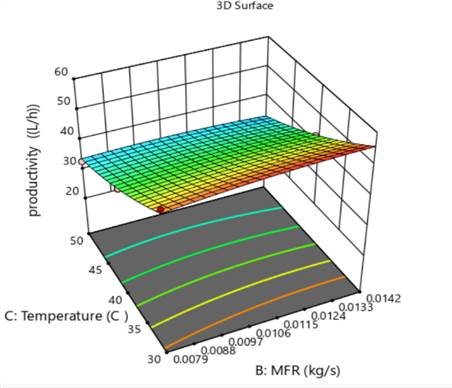


e


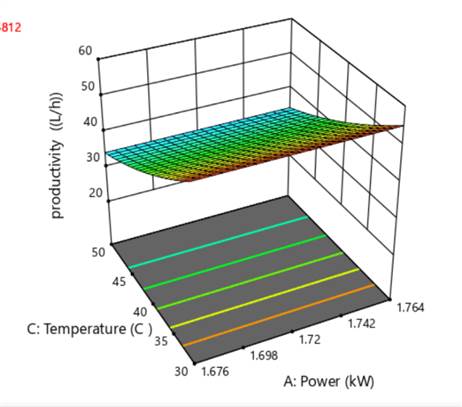


d

**(˚C)**

**f**

d

**(˚C)**

**Fig. S 5 |** Response surface plots showing the effect of independent factors on Pr (L/h), (a, b, c): Interactions between independent factors for OJ, (f, e, d): Interactions between independent factors for DBLJ

**Fig. S 6 |** Continuous-use simulation for temperature and acoustic intensity of CTS.

MATLAB code for continuous-use simulation for temperature and acoustic intensity of CTS:

% Example data

time = linspace(0, 8, 100); % time in hours

temperature = 25 + 10 * sin(pi * time / 8); % example temperature data

acousticIntensity = exp(-time); % example acoustic intensity data

% Use plotyy for dual y-axis plots

[ax, h1, h2] = plotyy(time, temperature, time, acousticIntensity);

% Customize plots

xlabel('Time (hours)')

ylabel(ax(1), 'Temperature (°C)')

ylabel(ax(2), 'Acoustic Intensity (a.u.)')

title('Ultrasonic Pasteurizer Heat and Acoustic Intensity Simulation')

set(h1, 'LineWidth', 2, 'Color', 'b');

set(h2, 'LineStyle', '--', 'LineWidth', 1.5, 'Color', 'r');

% Set limits for axes

set(ax(1), 'YLim', [20 40]);

set(ax(2), 'YLim', [0 1]);

set(ax, 'XLim', [0 8]);

grid on


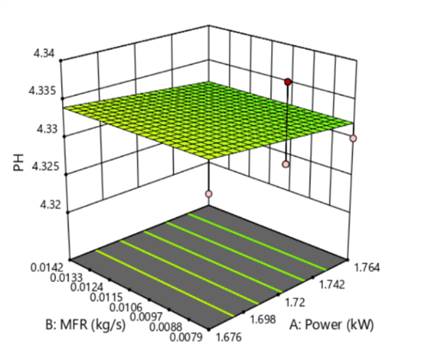


**a**


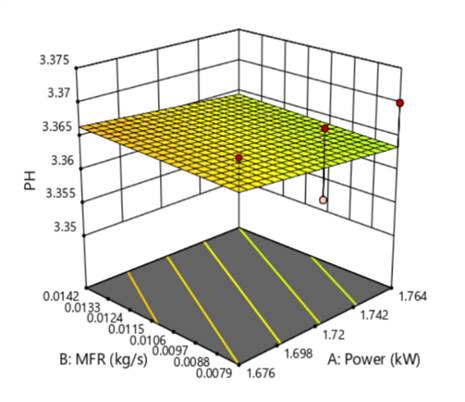


**b**

**Fig. S 7**: Response surface plots shows the effect of power and MFR on the pH, a: OJ, b: DBLJ.


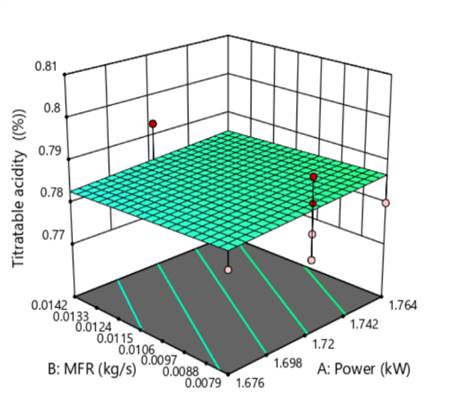

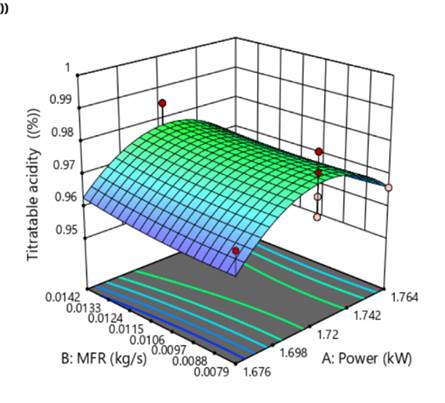


**b**

**a**

**Fig. S 8 |** Response surface plots shows the effect of power and MFR on the TA, a: OJ, b: DBLJ.

**b**


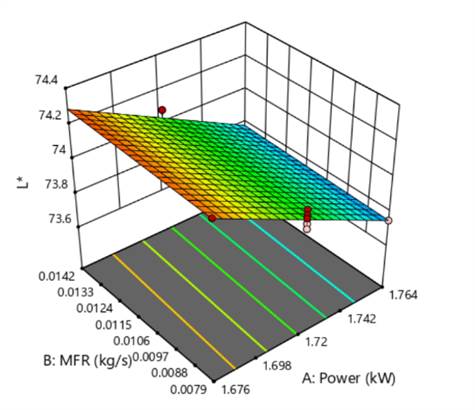

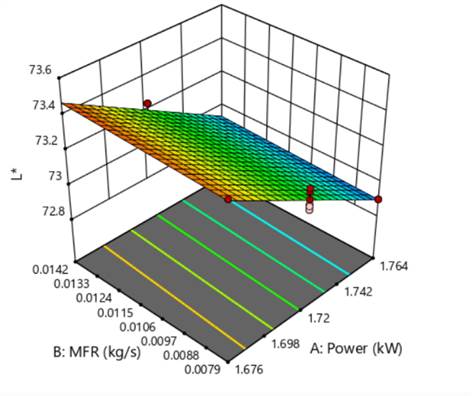


**a**

**Fig. S 9 |** Response surface plots shows the effect of power and MFR on the L*, a: OJ, b: DBLJ.


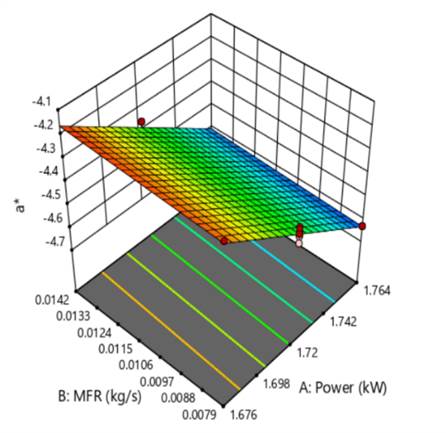

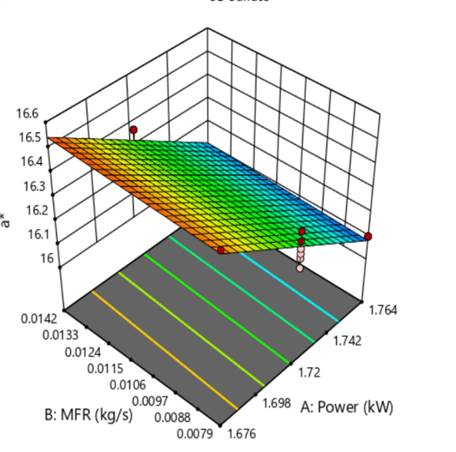


**a**

**b**

**Fig. S 10 |** Response surface plots shows the effect of power and MFR on the a*, a: OJ, b: DBLJ.


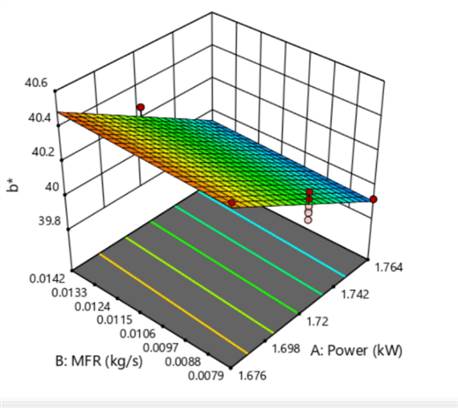

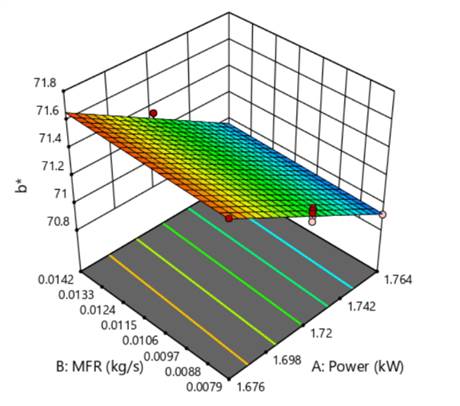


**a**

**b**

**Fig. S 11 |** Response surface plots shows the effect of power and MFR on the b*, a: OJ, b: DBLJ.


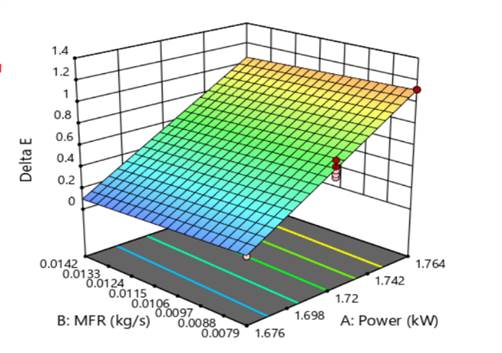

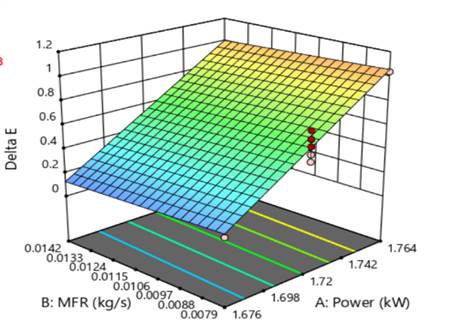


**b**

**a**

**ΔE**

**ΔE**

**Fig. S 12 |** Response surface plots shows the effect of power and MFR on the ΔE, a: OJ, b: DBLJ.


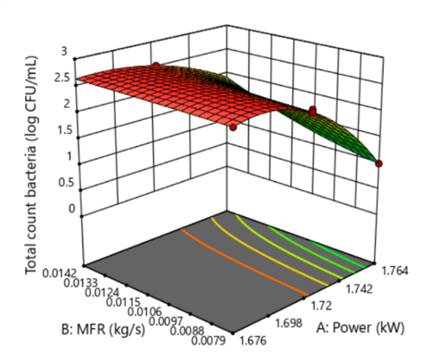


**a**


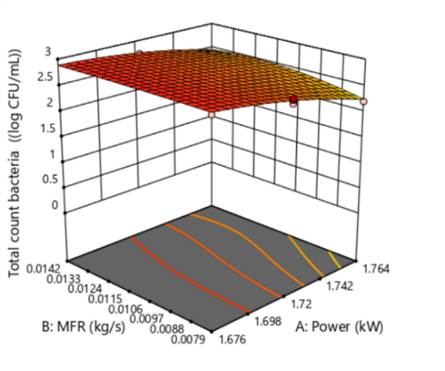


**d**


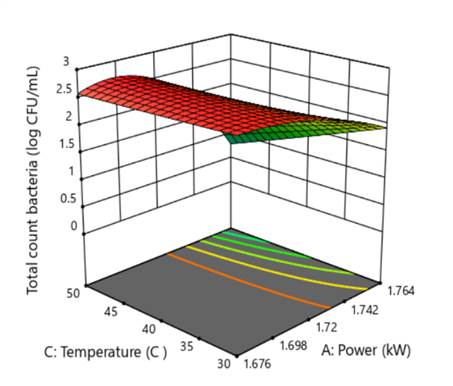


**b**


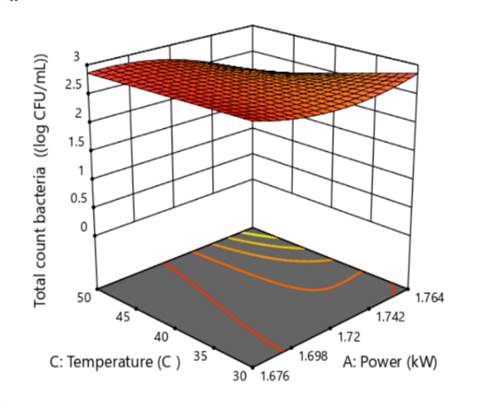


**e**

**C- Temperature (˚C)**


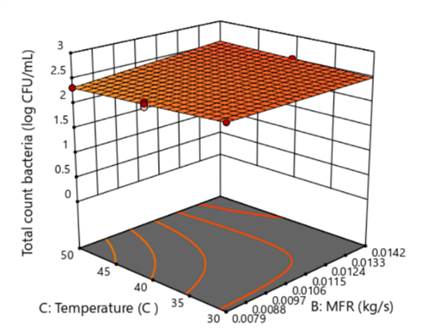


**c**

**C- Temperature (˚C)**


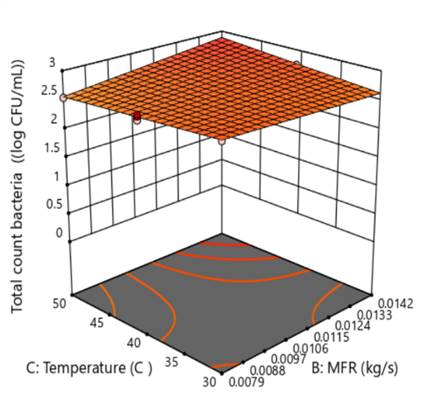


**f**

**C- Temperature (˚C)**

**Fig. S 13** **|** Response surface plots showing the effect of independent variables on TBC, (a,b and c): interactions between independent factors for OJ, (d, e and f): interactions between independent factors for DBLJ.

| **a** |
| --- |
| **b** |

**Fig. S 14** **|** Fit of the Weibull distribution model to the TPC survival curves of CTS-treated juices, a: OJ , b: DBLJ.


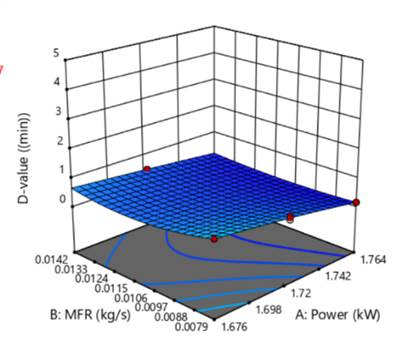

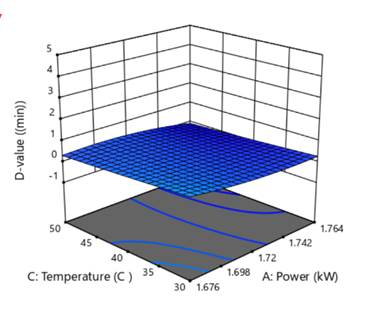

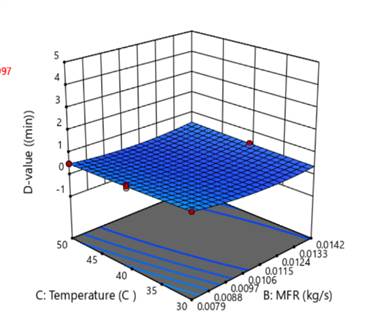


**a**

**c**

**b**


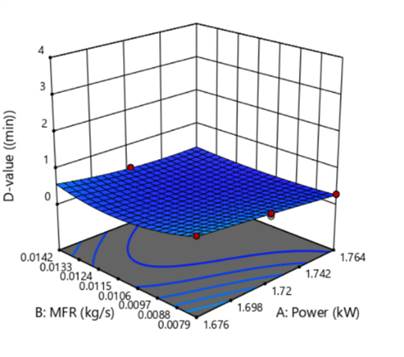

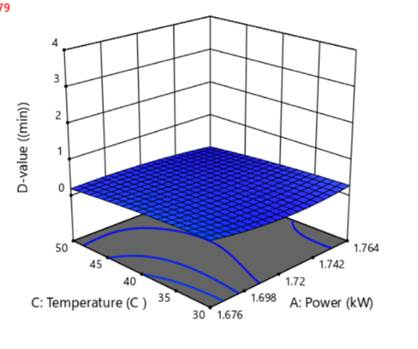

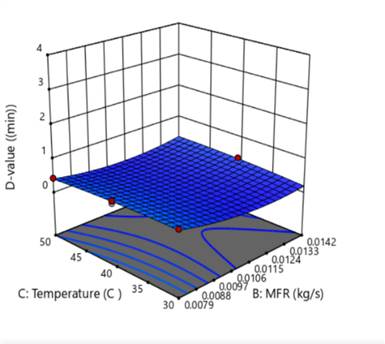


**f**

**e**

**d**

d

**Fig. S 15** **|** Response surface plots showing the effect of independent variables on D-value, (a,b and c): interactions between independent factors for OJ, (d, e and f): interactions between independent factors for DBLJ.

| **a**   | b   | c   |
| --- | --- | --- |
| d   | e   | f   |
| g   | h   | i   |
| j   | k    | l   |
| m   | n | O   |

**Fig. S16**: **|** Normal distribution plots of the dependent variables for OJ, a: SEC , b: EE , c: RT , d: Re, e: Pr , f: pH , g: TA , h: L* , i: a* , j: b* ,k: $\Delta E$ , l:PME activity, m: RA% , n: TBC , O: D-value.

| a   | b   | c   |
| --- | --- | --- |
| d   | e   | f   |
| g   | h   | i   |
| j   | k    | l   |
| m   | n | O   |

**Fig. S17** **|** Plots of residuals vs. predicted for the dependent variables of OJ, a: SEC, b: EE, c: RT, d: Re, e: Pr, f: pH, g: TA, h: L*, i: a*, j: b*, k: ∆E, l: PME activity, m: RA%, n: TBC, or: D value.

| **a**   | b   | c   |
| --- | --- | --- |
| d   | e   | f   |
| g   | h   | i   |
| j   | k   | l   |
| m   | n   | O   |

**Fig. S18** **|** Normal distribution plots of the dependent variables for DBLJ, a: SEC , b: EE , c: RT , d: Re, e: Pr , f: pH , g: TA , h: L* , i: a* , j: b* ,k: $\Delta E$ , l:PME activity, m: RA% , n: TBC , O: D-value.

| a   | b   | c   |
| --- | --- | --- |
| d   | e   | f   |
| g   | h   | i   |
| j   | k   | l   |
| m   | n   | O   |

**Fig. S19** **|** Plots of residuals vs. predicted for the dependent variables of DBLJ, a: SEC, b: EE, c: RT, d: Re, e: Pr, f: pH, g: TA, h: L*, i: a*, j: b*, k: ∆E, l: PME activity, m: RA%, n: TBC, or: D value.


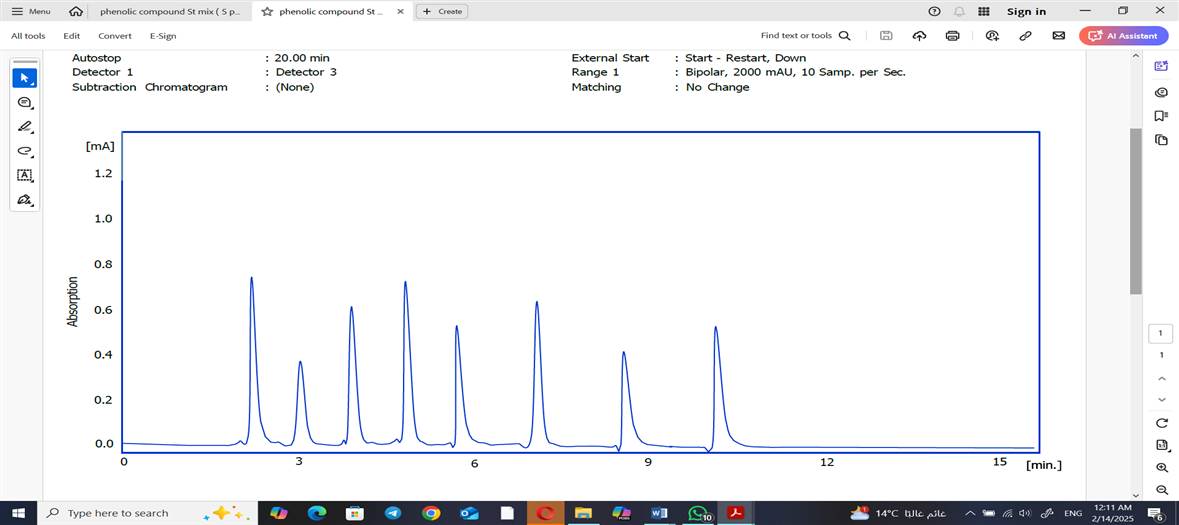


3.07

2.24

3.85

| Gallic acid |
| --- |

p-coumaric acid

Rutin

Apigenin

4.75

Naringenin

5.70

Ferulic acid

6.85

8.33

Hesperidin

Luteolin

9.93

**a**


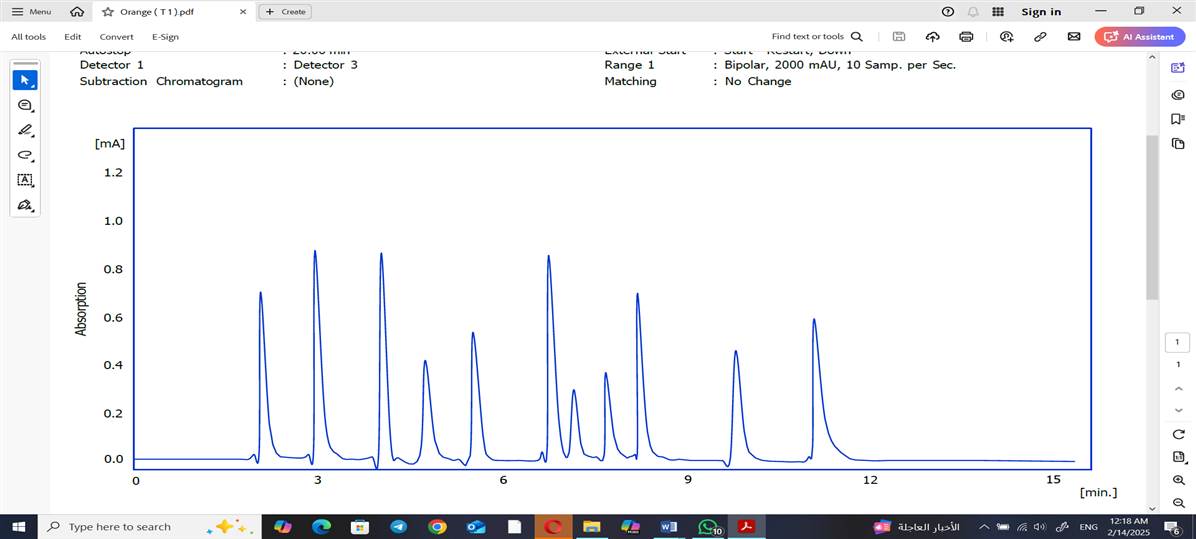


**b**

3.02

2.27

3.88

| Gallic acid |
| --- |

p-coumaric acid

Rutin

Apigenin

4.70

Naringenin

5.72

Ferulic acid

6.90

8.35

Hesperidin

Luteolin

7.80

9.90

11.25

7.35


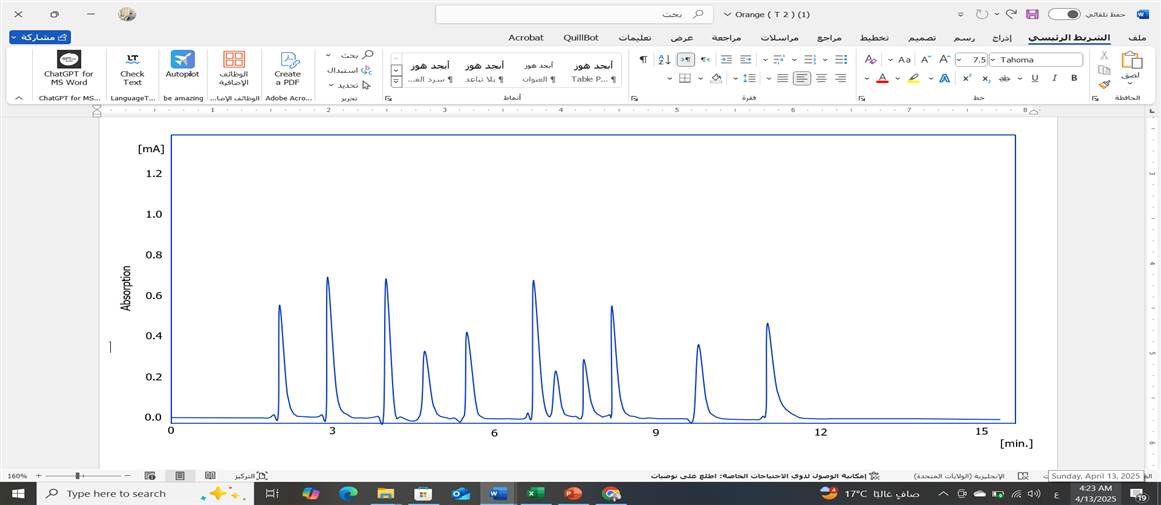


3.04

2.26

3.84

| Gallic acid |
| --- |

p-coumaric acid

Rutin

Apigenin

4.70

Naringenin

5.71

Ferulic acid

6.89

8.36

Hesperidin

Luteolin

7.85

9.93

11.76

7.33

**c**

**c**


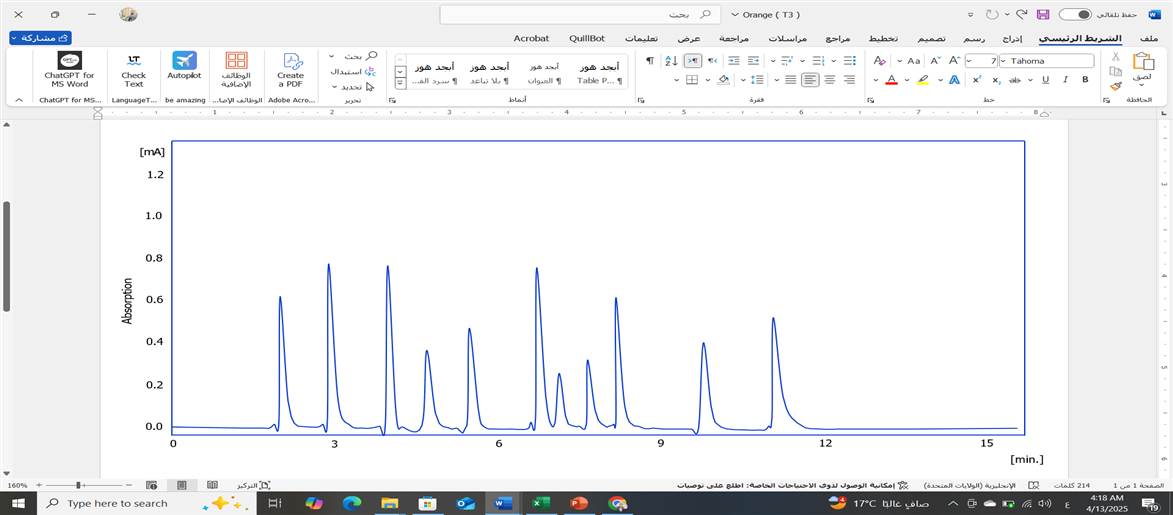


3.01

2.25

3.85

| Gallic acid |
| --- |

p-coumaric acid

Rutin

Apigenin

4.71

Naringenin

5.73

Ferulic acid

6.89

8.33

Hesperidin

Luteolin

7.83

9.91

11.22

7.31

**d**

**Fig. S 20 |** HPLC chromatograms of OJ: a: standard mixture, b: CTS-OJ, c: TP, d: Frish.


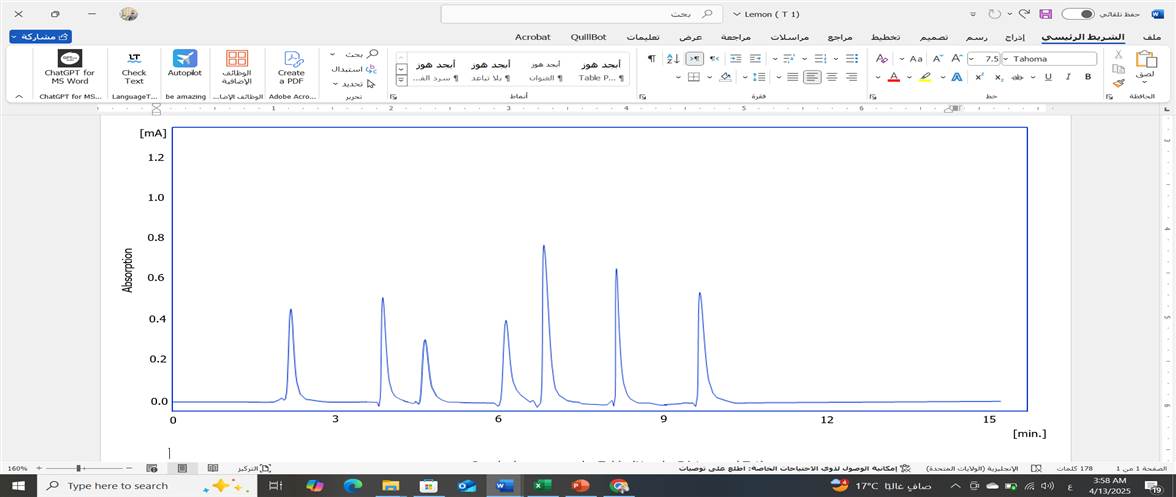


2.22

3.83

| Gallic acid |
| --- |

Rutin

Apigenin

4.72

Ferulic acid

6.87

8.33

Hesperidin

Luteolin

9.95

6.23

**a**


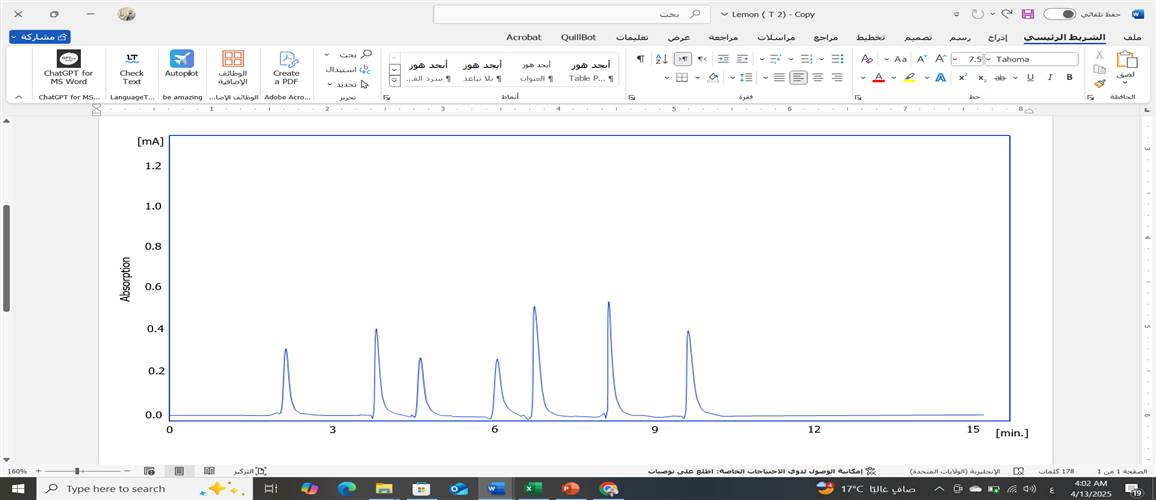


2.23

3.82

| Gallic acid |
| --- |

Rutin

Apigenin

4.73

6.85

8.31

Hesperidin

Luteolin

9.95

Ferulic acid

6.23

**b**


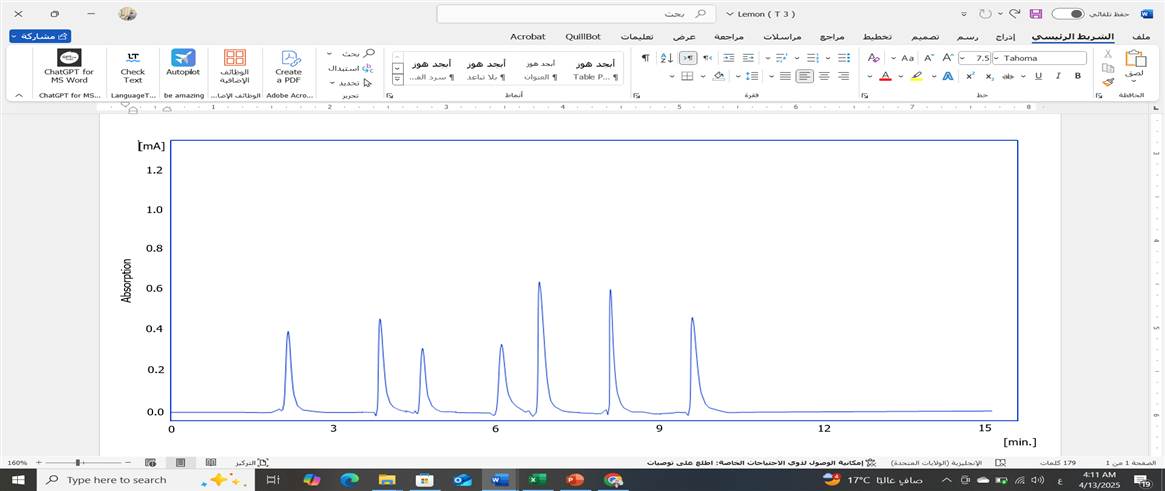


2.23

3.82

| Gallic acid |
| --- |

Rutin

Apigenin

4.73

6.85

8.31

Hesperidin

Luteolin

9.95

Ferulic acid

6.23

**c**

**Fig. S 21 |**  HPLC chromatograms of DBLJ: a: CTS-DBLJ , b:TP c: Frish.

1. **|** **Continuous Thermosonication System and it is mechanism of action**

The CTS system shown in Fig.S1 consists of five basic units. The first unit is the heating unit. This unit consists of a water bath (1), made in Germany, inside which is placed the juice container (4) made of stainless steel with a volume of 4 L. The system is also equipped with a pumping unit, consisting of a Taiwanese-made Upright diaphragm pump (9) (Model up 7100, 24 VDC, 29 W, pressure PAR 7.6, discharge rate 0.6 L/min), a plastic pipe (8), several connectors, and a plastic valve (10) for regulating the mass flow rate. The system is also equipped with a heat exchange unit. This unit consisted of a 5-L plastic reservoir (12) filled with cold water and ice cubes and equipped with a submersible pump (SP) (16) (China, AC 220 V, 50 Hz, discharge rate 1000 L/h, power 20 W). The SP transferred cold water through a 0.2 cm internal diameter plastic tube (14) to a stainless steel 316 coil heat exchanger (18) with a 0.6 cm internal diameter submerged in the ultrasonic bath. The hot water returns from the other end of the heat exchanger to the same tank through a plastic pipe (15) with an internal diameter of 0.2 cm. The heat exchange unit is controlled via a thermocouple (24) (inside the ultrasonic basin) connected to a digital temperature controller (26) via the control unit (25), through which the water temperature inside the US device basin is regulated to the treatment temperature. The pasteurization unit consists of a basin ultrasonic device (20), (LUC-405 Model, Volt 220V/50Hz) and a heat-resistant plastic tube (19), with an inner diameter of 0.2 cm and a length of 40 m, rolled into an oval shape and placed inside the basin of the ultrasonic device. This unit is also equipped with a thermocouple (23) connected to a digital gauge (27) to measure the temperature of the outgoing juice, as well as a control valve (21) to regulate the juice flow. The system is also equipped with a control and electrical power supply unit (25). This unit consists of an AC power source (32), switches and electrical wires, as well as a digital meter for measuring both current and voltage (28). The unit is also equipped with two thermocouples connected to two digital temperature controllers (6, 7) of Chinese origin, model STC-1000. The digital controller (6) operates via switch (2). This controller is designated to measure the temperature of the juice inside the plastic tube, while the other digital meter (7) operates via switch (3). This controller is designated to measure the temperature of the water inside the US device basin via a thermocouple, as well as to control the heat exchange unit. This unit is also equipped with a switch to operate the pump (4) and another switch to operate the US device (5). The first step to operate the CTS system involves connecting the electric current is connected to the power supply unit shown in Fig.S1 via switch (33). subsequently, switches (31) and (32) are operated, which are responsible for the digital controllers (26, 27) to measure and regulate the temperature inside the system. Then, the basin ultrasonic device is operated via switch (29). The water temperature inside the basin is adjusted via the control panel (22) in the ultrasonic device to the treatment temperature (30, 40, 50) °C. At the same time, the water bath (1) is operated to raise the juice temperature to the treatment temperature. Then, the required power (1.676, 1.72, 1.764) KW is determined via the control unit in the ultrasonic device (22). Then, the MFR (0.0142, 0.0079 and 0.0016) appropriate for each treatment is determined via valve (10). Finally, the pump (9) is operated via switch (30) through which the juice is pumped from the tank (4) and through the plastic tube (8) to the pasteurization unit (19). Then the juice will be exposed to the effect of ultrasonic waves directly from the wall of the basin to the tube and indirectly through the water surrounding the tube. The rise in temperature during the operation of the ultrasonic waves is controlled via the heat exchange unit. After that, the treated juice is filled into sterile bottles via the juice outlet valve (21) and kept at a temperature of 5±2 °C until the tests are carried out.

**2 |** **CTS system cleaning**

The cleaning process of the system was carried out according to the Cleaning–In-Place method. The process consists of filling the juice container with a cleaning solution and pumping it to the pasteurization unit using an electric pump to remove organic juice residues. After that, hot water and a disinfectant solution (sodium hypochlorite 50 ppm) are pumped in. Finally, the pasteurization unit is washed with hot water while using an ultrasonic device to sterilize and get rid of chemical residues in the pumping and pasteurization unit.
