# Supplementary material for: Optimized Continuous Thermosonication for Sustainable Pasteurization of Orange and Dried Black Lime Juices: Techno‐Functional, Physicochemical, and Microbial Assessment
Source: Food Sci Nutr. 2025 Nov 9;13(11):e71171. doi: 10.1002/fsn3.71171 (PMC12597978; doi:10.1002/fsn3.71171)
Supplement: Supplementary file 1 — Data S1: fsn371171‐sup‐0001‐Supinfo.zip. [file FSN3-13-e71171-s001.zip › fsn371171-sup-0002-Tables .docx]

| Source | SEC | | EE | | RT | | Re | | Pr | |
| --- | --- | --- | --- | --- | --- | --- | --- | --- | --- | --- |
|  | **Sum of Squares** | **p-value** | **Sum of Squares** | **p-value** | **Sum of Squares** | **p-value** | **Sum of Squares** | **p-value** | **Sum of Squares** | **p-value** |
| Model type | **QM** | **-** | **QM** | **-** | **RCM** | **-** | **LM** | **-** | **RQuM** | - |
| model | 59297.29 | **< 0.0001** | 870.11 | **< 0.0001** | 5.04 | **< 0.0001** | 6.69E+07 | **< 0.0001** | 1020.08 | **< 0.0001** |
| A-Power | 400.81 | **0.0016** | 34.1 | **< 0.0001** | 9.71E-10 | **0.6639** | 11196.81 | **0.181** | 1.28E-06 | **0.0734** |
| B-MFR | 310.32 | **0.0037** | 0.007 | **0.4793** | 0.0182 | **< 0.0001** | 6.68E+07 | **< 0.0001** | 0.7614 | **< 0.0001** |
| C-Temperature | 5740.97 | **< 0.0001** | 628.8 | **< 0.0001** | 3.72E-10 | **0.7871** | 59849.1 | **0.0052** | 213.12 | **< 0.0001** |
| AB | 1190.86 | **< 0.0001** | 0.0012 | **0.7622** | 2.44E-09 | **0.4968** | - | - | 2.59E-06 | **0.0513** |
| AC | 0 | **1** | 0.5375 | **< 0.0001** | 1.34E-08 | **0.1406** | - | - | 3.30E-07 | **0.321** |
| BC | 0 | **1** | 0.0055 | **0.5291** | 2.72E-07 | **0.0003** | - | - | 24.96 | **< 0.0001** |
| A² | 0.3571 | **0.9009** | 0.06 | **0.0563** | 1.88E-10 | **0.8474** | - | - | 0.0816 | **< 0.0001** |
| B² | 6605.1 | **< 0.0001** | 0.0003 | **0.8793** | 0.6695 | **< 0.0001** | - | - | 13.07 | **< 0.0001** |
| C² | 9.82 | **0.518** | 1.15 | **< 0.0001** | 1.03E-08 | **0.188** | - | - | 12.35 | **< 0.0001** |
| ABC | - | - | - | - | 7.03E-08 | **0.0081** | - | - | - | **-** |
| A^2^B | - | - | - | - | 3.73E-08 | **0.03** | - | - | 0.0121 | **< 0.0001** |
| A^2^C | - | - | - | - | 2.84E-08 | **0.0486** | - | - | 2.67 | **< 0.0001** |
| AB^2^ | - | - | - | - | 1.39E-08 | **0.1353** | - | - | - | **-** |
| A2B2 | - | - | - | - | - | - |  | - | 0.0968 | **< 0.0001** |
| Residual | 218.72 | - | 0.1288 | - | 2.79E-08 | - | 91578.12 | - | 2.03E-06 |  |
| Lack of Fit | 154.51 | **0.1786** | 0.025 | **0.928** | 5.49E-09 | **0.319** | 56535.03 | **0.6905** | 8.13E-07 | **0.2776** |
| Pure Error | 64.2 | - | 0.1039 | - | 2.25E-08 | - | 35043.09 | - | 1.21E-06 | - |
| Cor Total | 59516.01 | - | 870.24 | - | 5.04 | - | 6.70E+07 | - | 1020.08 | - |
| Std. Dev. | 4.68 | - | 0.1135 | - | 0.0001 | - | 75.65 | - | 0.0005 | - |
| Mean | 173.222 | - | 81.73 | - | 0.5291 | - | 3210.08 | - | 41.78 | - |
| C.V. % | 2.71 | - | 0.1389 | - | 0.0129 | - | 2.36 | - | 0.0013 | - |
| R² | **0.99** | **-** | **0.99** | **-** | **0.99** | **-** | **0.99** | **-** | **0.99** | - |
| Adjusted R² | **0.99** | **-** | **0.99** | - | **0.99** | - | **0.99** | - | **0.99** | - |
| Predicted R² | **0.98** | **-** | **0.99** | **-** | **0.99** | **-** | **0.99** | **-** | **0.99** | **-** |
| Adeq Precision | **61.5715** | **-** | **280.07** | - | **21687.96** | - | **159.312** | - | **60011.55** | - |

**Table S 1 |** ANOVA for QM, RCM, LM, and RQuM models and their effect on the dependent variables of OJ : SEC, EE, Re, RT, and Pr.

A: Power, B: MFR, C: Temperature, QM : Quadratic model , RCM ; Reduced Cubic model , LM: Linear model, and RQuM: Reduced Quartic model,

**Table S 2 |** ANOVA for RQuM, QM, LM, and RQM models and their effect on the dependent variables of DBLJ: SEC, EE, Re, RT, and Pr.

| Source | SEC | | EE | | RT | | Re | | Pr | |
| --- | --- | --- | --- | --- | --- | --- | --- | --- | --- | --- |
|  | **Sum of Squares** | **p-value** | **Sum of Squares** | **p-value** | **Sum of Squares** | **p-value** | **Sum of Squares** | **p-value** | **Sum of Squares** | **p-value** |
| Model type | **RQuM** | - | **QM** | **-** | **QM** | **-** | **LM** | **-** | **RQM** | **-** |
| model | 62007.61 | **< 0.0001** | 513.54 | **< 0.0001** | 5.11 | **< 0.0001** | 5.44E+07 | **< 0.0001** | 1129.85 | **< 0.0001** |
| A-Power | 11.29 | **< 0.0001** | 31.2 | **< 0.0001** | 2.66E-08 | **0.8053** | 2.85 | **0.979** | 1.48E-06 | **0.9983** |
| B-MFR | 28.16 | **< 0.0001** | 0.0929 | **0.0753** | 0.0346 | **< 0.0001** | 5.44E+07 | **< 0.0001** | 3.25 | **0.0085** |
| C-Temperature | 1921.51 | **< 0.0001** | 321.77 | **< 0.0001** | 2.08E-06 | **0.052** | 26923.05 | **0.0195** | 819.06 | **< 0.0001** |
| AB | 8.14 | **< 0.0001** | 0.0603 | **0.1409** | 5.16E-08 | **0.7315** | - | **-** | 1.92E-06 | **0.9981** |
| AC | 0 | **1** | 0.4628 | **0.0013** | 1.36E-08 | **0.8597** | - | **-** | 1.86E-06 | **0.9981** |
| BC | 0 | **1** | 0.079 | **0.0971** | 7.06E-06 | **0.0021** | - | **-** | 27.8 | **< 0.0001** |
| A² | 83.65 | **< 0.0001** | 0.0753 | **0.1043** | 4.81E-08 | **0.7405** | - | **-** | - | **-** |
| B² | 4992.86 | **< 0.0001** | 0.044 | **0.202** | 0.6774 | **< 0.0001** | - | **-** | 30.64 | **< 0.0001** |
| C² | 7.62 | **< 0.0001** | 32.99 | **< 0.0001** | 7.41E-07 | **0.2109** | - | **-** | 8.82 | **0.0003** |
| ABC | - | **-** | - | **-** |  |  | - | **-** | - | **-** |
| A^2^B | 112.21 | **< 0.0001** | - | **-** |  |  | - | **-** | - | **-** |
| A^2^C | 0 | **1** | - | **-** |  |  | - | **-** | - | **-** |
| AB^2^ | 151.47 | **< 0.0001** | - | **-** |  |  | - | **-** | - | **-** |
| A^2^B^2^ | 35.73 | **< 0.0001** | - | **-** |  | **-** | - | **-** | - | **-** |
| Residual | 0.0029 | **-** | 0.2359 | **-** | 4.15E-06 | **-** | 63941.9 | **-** | 3.5 | **-** |
| Lack of Fit | 0 | **1** | 0.18 | **-** | 2.91E-06 | **0.1856** | 28255.18 | **0.9267** | 3.49 | **< 0.0001** |
| Pure Error | 0.0029 | **-** | 0.0559 | **-** | 1.24E-06 | **-** | 35686.72 | **-** | 0.0001 | **-** |
| Cor Total | 62007.61 | **-** | 513.78 | **-** | 5.11 | **-** | 5.45E+07 | **-** | 1133.35 | **-** |
| Std. Dev. | 0.0221 | **-** | 0.1536 | **-** | 0.0006 | **-** | 63.22 | **-** | 0.5637 | **-** |
| Mean | 177.5 | **-** | 78.92 | **-** | 0.5328 | **-** | 2919.65 | **-** | 40.95 | **-** |
| C.V. % | 0.0125 | **-** | 0.1946 | **-** | 0.1209 | **-** | 2.17 | **-** | 1.38 | **-** |
| R² | **0.99** | **-** | **0.99** | **-** | **0.99** | **-** | **0.99** | **-** | **0.97** | **-** |
| Adjusted R² | **0.99** | **-** | **0.99** | **-** | **0.99** | **-** | **0.99** | **-** | **0.99** | **-** |
| Predicted R² | **NA⁽¹⁾** | **-** | **0.99** | **-** | **0.98** | **-** | **0.99** | **-** | **0.98** | **-** |
| Adeq Precision | **11702.12** | **-** | **158.33** | **-** | **2742.3405** | **-** | **168.67** | **-** | **72.40** | **-** |

RQM: Reduced Quadratic model ,

**Table S 3 |** Regression coefficients of the mathematical models used to predict the physical properties of the CTS performance for OJ and DBLJ.

|  | OJ | | | | | DBLJ | | | | |
| --- | --- | --- | --- | --- | --- | --- | --- | --- | --- | --- |
|  | SEC | EE | RT | Re | Pr | SEC | EE | RT | Re | Pr |
| intercept | -1267.1859 | 304.173 | 0.64227 | 1587.018 | 71.16 | -5610.07013 | 300.2274 | 2.03327 | 224.20 | 86.56358 |
| A | 824.3904 | -287.488 | 1.39401 | -760.492 | -0.016 | 5951.65497 | -321.636 | -0.226643 | -12.13 | -0.07337 |
| B | 1.97E+05 | -90.9759 | -257.55462 | 4.10E+05 | 701.89 | 3.51E+06 | -639.403 | -295.36407 | 3.70E+05 | 3087.215 |
| C | 2.06242 | 2.43507 | 0.019607 | -7.73622 | -0.87 | 1.39227 | 4.34342 | -0.000037 | -5.19 | -2.04021 |
| AB | -1.31E+05 | 45.0251 | -43.01819 | - | - | -3.98E+06 | 313.2623 | -0.289837 | - | 1.76529 |
| AC | 1.83 | -0.58912 | -0.023184 | - | - | -2.35E-09 | -0.54664 | -0.000094 | - | 0.001096 |
| BC | -2.47E-10 | 0.41533 | 0.055257 | - | - | 2.51E-10 | 1.57778 | -0.014914 | - | -29.5873 |
| A^2^ | 1.01909 | 76.2999 | -0.408287 | - | - | -1491.17737 | 85.47842 | 0.068297 | - | - |
| B^2^ | -8.29E+06 | 268.591 | 12340.36 | - | - | -3.57E+08 | 3186.351 | 12505.17174 | - | -77965.1 |
| C^2^ | -0.02578 | -0.00647 | -6.11E-07 | - | - | 0.022534 | -0.03464 | 5.19091E-06 | - | 0.016606 |
| ABC | 1.48E-10 | - | -0.033828 | - | - | - | - | - | - | - |
| A^2^B | 2.41E-08 | - | 12.51152 | - | - | 1.12E+06 | - | - | - | - |
| A^2^C | 2.12E-11 | - | 0.006879 | - | - | 6.94E-10 | - | - | - | - |
| AB^2^ | 5.53E+06 | - | 53.28138 | - | - | 4.11E+08 | - | - | - | - |
| A^2^B^2^ | - | - |  | - | - | -1.18E+08 | - | - | - | - |

**Table S 4** **|** CCD matrix for the effect of power, MFR, and temperature on the physical properties of OJ and DBLJ treated with CTS.

|  | Independent variables | | | | Dependent variables | | | | | | | | | | | |
| --- | --- | --- | --- | --- | --- | --- | --- | --- | --- | --- | --- | --- | --- | --- | --- | --- |
|  | |  |  |  | **OJ** | | | | | | **DBLJ** | | | | | |
| RUN | | **P** | $\mathbf{MFR}$ | **T** | **pH** | **TA (%)** | **L*** | **a*** | **b*** | **ΔE** | **pH** | **TA (%)** | **L*** | **a*** | **b*** | **ΔE** |
|  | | 1.72 | 0.0142 | 40 | 4.33 | 0.793 | 74.08 | -4.36 | 71.35 | 0.51 | 3.36 | 0.985 | 73.24 | 16.37 | 40.27 | 0.48 |
|  | | 1.72 | 0.0079 | 50 | 4.33 | 0.8 | 73.89 | -4.52 | 71.09 | 0.87 | 3.35 | 0.998 | 73.03 | 16.2 | 40.05 | 0.83 |
|  | | 1.676 | 0.0142 | 30 | 4.34 | 0.774 | 74.33 | -4.14 | 71.7 | 0.03 | 3.37 | 0.96 | 73.52 | 16.58 | 40.54 | 0.04 |
|  | | 1.72 | 0.0079 | 40 | 4.33 | 0.793 | 74.03 | -4.39 | 71.31 | 0.58 | 3.36 | 0.972 | 73.21 | 16.32 | 40.21 | 0.56 |
|  | | 1.764 | 0.0142 | 30 | 4.33 | 0.787 | 73.83 | -4.57 | 71.01 | 0.98 | 3.36 | 0.979 | 73.01 | 16.16 | 40.01 | 0.89 |
|  | | 1.676 | 0.0016 | 50 | 4.33 | 0.787 | 74.17 | -4.25 | 71.51 | 0.3 | 3.36 | 0.979 | 73.34 | 16.46 | 40.37 | 0.32 |
|  | | 1.72 | 0.0079 | 30 | 4.34 | 0.78 | 74.12 | -4.31 | 71.42 | 0.42 | 3.37 | 0.972 | 73.27 | 16.4 | 40.3 | 0.43 |
|  | | 1.764 | 0.0016 | 30 | 4.34 | 0.774 | 73.83 | -4.61 | 70.97 | 1.03 | 3.37 | 0.966 | 72.95 | 16.13 | 39.98 | 0.96 |
|  | | 1.764 | 0.0016 | 50 | 4.33 | 0.8 | 73.65 | -4.68 | 70.83 | 1.26 | 3.36 | 0.998 | 72.81 | 16.02 | 39.82 | 1.2 |
|  | | 1.764 | 0.0142 | 50 | 4.32 | 0.793 | 73.69 | -4.66 | 70.88 | 1.19 | 3.36 | 0.972 | 72.84 | 16.05 | 39.86 | 1.14 |
|  | | 1.72 | 0.0079 | 40 | 4.34 | 0.774 | 74.01 | -4.41 | 71.29 | 0.62 | 3.36 | 0.985 | 73.17 | 16.29 | 40.17 | 0.63 |
|  | | 1.72 | 0.0079 | 40 | 4.34 | 0.78 | 73.98 | -4.42 | 71.27 | 0.65 | 3.36 | 0.972 | 73.14 | 16.25 | 40.14 | 0.68 |
|  | | 1.72 | 0.0079 | 40 | 4.33 | 0.787 | 74.06 | -4.4 | 71.33 | 0.56 | 3.36 | 0.979 | 73.13 | 16.27 | 40.13 | 0.69 |
|  | | 1.72 | 0.0016 | 40 | 4.32 | 0.806 | 73.91 | -4.49 | 71.15 | 0.81 | 3.36 | 0.979 | 73.08 | 16.23 | 40.09 | 0.76 |
|  | | 1.676 | 0.0142 | 50 | 4.34 | 0.774 | 74.22 | -4.21 | 71.57 | 0.21 | 3.37 | 0.972 | 73.4 | 16.5 | 40.42 | 0.23 |
|  | | 1.764 | 0.0079 | 40 | 4.33 | 0.78 | 73.75 | -4.63 | 70.92 | 1.12 | 3.37 | 0.966 | 72.9 | 16.1 | 39.92 | 1.04 |
|  | | 1.72 | 0.0079 | 40 | 4.33 | 0.793 | 73.95 | -4.45 | 71.23 | 0.71 | 3.37 | 0.966 | 73.23 | 16.36 | 40.25 | 0.51 |
|  | | 1.676 | 0.0016 | 30 | 4.34 | 0.774 | 74.3 | -4.16 | 71.66 | 0.08 | 3.37 | 0.96 | 73.48 | 16.56 | 40.51 | 0.09 |
|  | | 1.72 | 0.0079 | 40 | 4.34 | 0.774 | 74.02 | -4.38 | 71.3 | 0.59 | 3.37 | 0.966 | 73.11 | 16.21 | 40.09 | 0.75 |
|  | | 1.676 | 0.0079 | 40 | 4.33 | 0.78 | 74.26 | -4.18 | 71.62 | 0.14 | 3.37 | 0.966 | 73.44 | 16.53 | 40.48 | 0.15 |

**Table S 5 |** ANOVA for LM and their effect on the dependent variables of OJ: pH, TA, L*, a*, b* and ΔE

| Source | pH | | TA | | L* | | a* | | b* | | ΔE | |
| --- | --- | --- | --- | --- | --- | --- | --- | --- | --- | --- | --- | --- |
|  | **Sum of Squares** | **p-value** | **Sum of Squares** | **p-value** | **Sum of Squares** | **p-value** | **Sum of Squares** | **p-value** | **Sum of Squares** | **p-value** | **Sum of Squares** | **p-value** |
| Model type | **LM** | **-** | **LM** | **-** | **LM** | **-** | **LM** | **-** | **LM** | **-** | **LM** | **-** |
| model | 0.0004 | **0.0596** | 0.001 | **0.0633** | 0.7109 | **< 0.0001** | 0.5228 | **< 0.0001** | 1.28 | **< 0.0001** | 2.51 | **< 0.0001** |
| A-Power | 1.00E-05 | **0.5593** | 0 | **0.621** | 0.6401 | **< 0.0001** | 0.4884 | **< 0.0001** | 1.19 | **< 0.0001** | 2.32 | **< 0.0001** |
| B-MFR | 5.42E-20 | **1** | 0 | **0.5946** | 0.0084 | **0.0205** | 0.0063 | **0.0188** | 0.0152 | **0.0163** | 0.0299 | **0.0147** |
| C-Temperature | 0.0004 | **0.0025** | 0.0009 | **0.067** | 0.0624 | **< 0.0001** | 0.0281 | **< 0.0001** | 0.0774 | **< 0.0001** | 0.1668 | **< 0.0001** |
| Residual | 0.0004 | **-** | 0.0011 |  | 0.0204 |  | 0.0146 |  | 0.0338 |  | 0.0641 | **-** |
| Lack of Fit | 0.0003 | **0.5865** | 0.0007 | **0.6497** | 0.0129 | **0.6598** | 0.0115 | **0.29** | 0.0277 | **0.2178** | 0.0488 | **0.3593** |
| Pure Error | 0.0002 | - | 0.0004 | - | 0.0075 | - | 0.0031 | - | 0.0061 | - | 0.0153 | - |
| Cor Total | 0.0008 | - | 0.002 | - | 0.7313 | - | 0.5374 | - | 1.32 | - | 2.58 | - |
| Std. Dev. | 0.0053 | - | 0.0082 | - | 0.0357 | - | 0.0302 | - | 0.046 | - | 0.0633 | - |
| Mean | 4.33 | - | 0.7854 | - | 74 | - | -4.41 | - | 71.27 | - | 0.6334 | - |
| C.V. % | 0.1224 | - | 1.04 | - | 0.0482 | - | 0.6855 | - | 0.0645 | - | 9.99 | - |
| R² | **0.45** | **-** | **0.48** | **-** | **0.97** | **-** | **0.97** | **-** | **0.97** | **-** | **0.98** | **-** |
| Adjusted R² | **0.35** | **-** | **0.38** | **-** | **0.97** | **-** | **0.97** | **-** | **0.97** | **-** | **0.97** | **-** |
| Predicted R² | **0.22** | **-** | **0.28** | **-** | **0.96** | **-** | **0.96** | **-** | **0.96** | **-** | **0.96** | **-** |
| Adeq Precision | **5.90** | **-** | **6.80** | **-** | **45.25** | **-** | **44.22** | **-** | **45.93** | **-** | **47.00** | **-** |

**Table S 6 |** ANOVA for LM, and RQuM models and their effect on the dependent variables of DBLJ: pH, TA, L*, a*, b*, and ΔE .

| Source | pH | | TA | | L* | | a* | | b* | | ΔE | |
| --- | --- | --- | --- | --- | --- | --- | --- | --- | --- | --- | --- | --- |
|  | **Sum of Squares** | **p-value** | **Sum of Squares** | **p-value** | **Sum of Squares** | **p-value** | **Sum of Squares** | **p-value** | **Sum of Squares** | **p-value** | **Sum of Squares** | **p-value** |
| Model type | **LM** | **-** | **RQuM** | **-** | **LM** | **-** | **LM** | - | **LM** | - | **LM** | **-** |
| model | 0.0003 | **0.057** | 0.0016 | **0.0876** | 0.7907 | **< 0.0001** | 0.5136 | < 0.0001 | 0.8234 | < 0.0001 | 2.13 | **< 0.0001** |
| A-Power | 1.00E-05 | **0.5481** | 0.0001 | **0.3449** | 0.7129 | **< 0.0001** | 0.4709 | < 0.0001 | 0.7453 | < 0.0001 | 1.93 | **< 0.0001** |
| B-MFR | 0 | **0.5481** | 0 | **0.7004** | 0.0123 | **0.008** | 0.0068 | 0.0476 | 0.0109 | 0.0351 | 0.0298 | **0.0238** |
| C-  Temperature | 0.0002 | **0.061** | 0.0003 | **0.0526** | 0.0656 | **< 0.0001** | 0.036 | 0.0002 | 0.0672 | < 0.0001 | 0.1686 | **< 0.0001** |
| AB | - | **-** | 0 | **0.5795** | - | **-** | - | - | - | - | - | **-** |
| AC | - | **-** | 1.25E-07 | **0.9657** | - | **-** | - | - | - | - | - | **-** |
| BC | - | **-** | 0.0002 | **0.1187** | - | **-** | - | - | - | - | - | **-** |
| A² | - | **-** | 0.0003 | **0.0567** | - | **-** | - | - | - | - | - | **-** |
| B² | - | **-** | 0 | **0.5985** | - | **-** | - | - | - | - | - | **-** |
| C² | - | **-** | 0.0001 | **0.2922** | - | **-** | - | - | - | - | - | **-** |
| A²B² | - | **-** | 8.29E-06 | **0.7271** | - | **-** | - | - | - | - | - | **-** |
| Residual | 0.0004 | **-** | 0.0006 | **-** | 0.0213 |  | 0.0256 |  | 0.0329 |  | 0.0765 |  |
| Lack of Fit | 0.0003 | **0.5415** | 0.0003 | **0.3753** | 0.0102 | **0.8954** | 0.0117 | 0.9147 | 0.0161 | 0.8823 | 0.0357 | **0.9063** |
| Pure Error | 0.0001 | **-** | 0.0003 | **-** | 0.0112 | **-** | 0.0139 | - | 0.0167 | - | 0.0408 | **-** |
| Cor Total | 0.0007 | **-** | 0.0022 | **-** | 0.8121 | **-** | 0.5393 | - | 0.8563 | - | 2.2 | **-** |
| Std. Dev. | 0.0052 | **-** | 0.008 | **-** | 0.0365 | **-** | 0.04 | - | 0.0453 | - | 0.0691 | **-** |
| Mean | 3.36 | **-** | 0.9742 | **-** | 73.17 | **-** | 16.3 | - | 40.18 | - | 0.6188 | **-** |
| C.V. % | 0.1532 | **-** | 0.8208 | **-** | 0.0499 | **-** | 0.2456 | - | 0.1128 | - | 11.17 | **-** |
| R² | **0.39** | **-** | **0.74** | **-** | **0.97** | **-** | **0.95** | **-** | **0.96** | **-** | **0.97** | **-** |
| Adjusted R² | **0.27** | **-** | **0.49** | **-** | **0.97** | **-** | **0.94** | **-** | **0.95** | **-** | **0.96** | **-** |
| Predicted R² | **0.085** | **-** | **NA⁽¹⁾** | **-** | **0.96** | **-** | **0.94** | **-** | **0.95** | **-** | **0.95** | **-** |
| Adeq Precision | **6.07** | **-** | **6.4416** | **-** | **46.89** | **-** | **33.85** | **-** | **38.28** | **-** | **40.33** | **-** |

**Table S 7 |** Regression coefficients for the mathematical models used to predict the physical properties of OJ and DBLJ treated with CTS

|  | OJ | | | | |  |  | DBLJ | | | | |  |
| --- | --- | --- | --- | --- | --- | --- | --- | --- | --- | --- | --- | --- | --- |
|  | **pH** | **TA** | **L*** | **a*** | **b*** | **ΔE** |  | **pH** | **TA** | **L*** | **a*** | **b*** | **ΔE** |
| intercept | 4.396 | 0.7 | 84.17 | 4.41 | 85.06 | -18.62 |  | 3.42 | -18.43 | 83.88 | 24.99 | 51.14 | -16.99 |
| A | -0.023 | 0.03 | -5.75 | -5.02 | -7.84 | 10.94 |  | -0.023 | 22.44 | -6.07 | -4.93 | -6.20 | 9.98 |
| B | 3.66  E-14 | -0.22 | 4.60 | 3.97 | 6.19 | -8.69 |  | 0.16 | 79.36 | 5.56 | 4.14 | 5.24 | -8.67 |
| C | -0.0006 | 0.001 | -0.0079 | -0.0053 | -0.0088 | 0.0129 |  | -0.0005 | -0.0042 | -0.0081 | -0.006 | -0.0082 | 0.012986 |
| AB | - | - | - | - | - | - |  | - | -45.37 | - | - | - | - |
| AC | - | - | - | - | - | - |  | - | 0.00028 | - | - | - | - |
| BC | - | - | - | - | - | - |  | - | -0.077 | - | - | - | - |
| A^2^ | - | - | - | - | - | - |  | - | -6.47 | - | - | - | - |
| B^2^ | - | - | - | - | - | - |  | - | -2060.64 | - | - | - | - |
| C^2^ | - | - | - | - | - | - |  | - | 0.00007 | - | - | - | - |
| A^2^B | - | - | - | - | - | - |  | - | 726.94 | - | - | - | - |

**Table S 8 |** ANOVA for LM, RQuM, and RCM models and their effect on the dependent variables of OJ: PEM, RA, TBC, and D-value

| Source –LIME | PME | | RA | | TCB | | D-value | |
| --- | --- | --- | --- | --- | --- | --- | --- | --- |
|  | **Sum of Squares** | **p-value** | **Sum of Squares** | **p-value** | **Sum of Squares** | **p-value** | **Sum of Squares** | **p-value** |
| Model type | **LM** | **-** | **LM** | **-** | **RQuM** | - | **RCM** | - |
| model | 1.45E-08 | **< 0.0001** | 5172.51 | **< 0.0001** | 8.09 | **< 0.0001** | 20.01 | **< 0.0001** |
| A-Power | 1.16E-08 | **< 0.0001** | 4164.68 | **< 0.0001** | 1.23 | **< 0.0001** | 0.1447 | **0.0002** |
| B-MFR | 2.00E-09 | **< 0.0001** | 696.11 | **< 0.0001** | 0.0127 | **0.0426** | 0.0285 | **0.0104** |
| C-Temperature | 8.71E-10 | **< 0.0001** | 311.72 | **< 0.0001** | 0.0011 | **0.4697** | 0.0007 | **0.582** |
| AB | **-** | **-** | **-** | **-** | 0.2523 | **< 0.0001** | 0.0365 | **0.006** |
| AC | **-** | **-** | **-** | **-** | 0.441 | **< 0.0001** | 0.001 | **0.5195** |
| BC | **-** | **-** | **-** | **-** | 0.12 | **0.0004** | 0.2607 | **< 0.0001** |
| A² | **-** | **-** | **-** | **-** | 0.4092 | **< 0.0001** | 0.0017 | **0.4064** |
| B² | **-** | **-** | **-** | **-** | 4.17E-06 | **0.9628** | 1.7 | **< 0.0001** |
| C² | **-** | **-** | **-** | **-** | 0.0001 | **0.8524** | 0.0012 | **0.4806** |
| ABC | **-** | **-** | **-** | **-** | 0.1058 | **0.0006** | 0.0015 | **0.4301** |
| A²B | **-** | **-** | **-** | **-** | 0.0461 | **0.0036** | 0.0021 | **0.3584** |
| A²C | **-** | **-** | **-** | **-** | 0.1277 | **0.0004** | 0.0503 | **0.0028** |
| AB² | **-** | **-** | **-** | **-** | 0.0792 | **0.0011** | 0.2428 | **< 0.0001** |
| A²B² | **-** | **-** | **-** | **-** | 0.0169 | **0.0263** |  | **-** |
| Residual | 4.05E-10 |  | 146.19 |  | 0.0087 | **-** | 0.0127 | **-** |
| Lack of Fit | 3.12E-10 | **0.3354** | 110.13 | **0.3782** | - | **-** | 0 | **0.9079** |
| Pure Error | 9.28E-11 | **-** | 36.06 | **-** | 0.0087 | **-** | 0.0126 | **-** |
| Cor Total | 1.49E-08 | **-** | 5318.7 | **-** | 8.1 | **-** | 20.02 | **-** |
| Std. Dev. | 5.03E-06 | **-** | 3.02 | **-** | 0.0417 | **-** | 0.046 | **-** |
| Mean | 0.0001 | **-** | 37.51 | **-** | 2.18 | **-** | 0.9715 | **-** |
| C.V. % | 8.03 | **-** | 8.06 | **-** | 1.91 | **-** | 4.73 | **-** |
| R² | **0.97** | **-** | **0.97** | **-** | **0.99** |  | **0.99** | **-** |
| Adjusted R² | **0.97** | **-** | **0.97** | **-** | **0.99** |  | **0.99** | **-** |
| Predicted R² | **0.95** | **-** | **0.95** | **-** | **NA⁽¹⁾** |  | **0.99** | **-** |
| Adeq Precision | **51.12** | **-** | **50.80** | **-** | **Adeq Precision** | **74.26** | **103.68** | **-** |

**Table S 9 |** ANOVA for LM, RQuM , and RCM models and their effect on the dependent variables of DBLJ: PEM, RA, TBC, and D-value .

| Source –LIME | PME | | RA | | TCB | | D-value | |
| --- | --- | --- | --- | --- | --- | --- | --- | --- |
|  | **Sum of Squares** | **p-value** | **Sum of Squares** | **p-value** | **Sum of Squares** | **p-value** | **Sum of Squares** | **p-value** |
| Model type | **LM** | **-** | **LM** | **-** | **RQuM** | - | **RCM** | - |
| model | 6.15E-09 | < 0.0001 | 4958.68 | < 0.0001 | 7.46 | < 0.0001 | 17.79 | < 0.0001 |
| A-Power | 5.02E-09 | < 0.0001 | 4048.23 | < 0.0001 | 0.1388 | < 0.0001 | 0.0202 | 0.0099 |
| B-MFR | 7.40E-10 | < 0.0001 | 608.15 | < 0.0001 | 0.0365 | 0.0022 | 0.0521 | 0.001 |
| C-Temperature | 3.89E-10 | < 0.0001 | 302.3 | < 0.0001 | 0.0009 | 0.5093 | 0 | 0.9177 |
| AB | **-** | **-** | **-** | **-** | 0.0145 | 0.0238 | 0.0131 | 0.0242 |
| AC | **-** | **-** | **-** | **-** | 0.1968 | < 0.0001 | 0.0003 | 0.6561 |
| BC | **-** | **-** | **-** | **-** | 0.3715 | < 0.0001 | 0.1857 | < 0.0001 |
| A² | **-** | **-** | **-** | **-** | 0.0004 | 0.6623 | 0.0047 | 0.123 |
| B^2^ | **-** | **-** | **-** | **-** | **-** | **-** | 1.64 | < 0.0001 |
| C^2^ | **-** | **-** | **-** | **-** | **-** | **-** | 0.0048 | 0.119 |
| ABC | **-** | **-** | **-** | **-** | 0.3503 | < 0.0001 | 0.0011 | 0.4209 |
| A2B | **-** | **-** | **-** | **-** | **-** | **-** | 0.0255 | 0.0059 |
| A²C | **-** | **-** | **-** | **-** | 0.0846 | 0.0001 | 0.027 | 0.0051 |
| AB² | **-** | **-** | **-** | **-** | 0.0847 | 0.0001 | 0.3437 | < 0.0001 |
| A²B² | **-** | **-** | **-** | **-** | 0.2455 | < 0.0001 | **-** | **-** |
| Residual | 2.18E-10 |  | 171.32 |  | 0.015 |  | 0.0088 | 0.0766 |
| Lack of Fit | 1.57E-10 | 0.4576 | 127.21 | 0.4049 | 0.0092 | 0.1605 | 0.0044 | **-** |
| Pure Error | 6.08E-11 | **-** | 44.1 | **-** | 0.0058 | **-** | 0.0044 | **-** |
| Cor Total | 6.37E-09 | **-** | 5130 | **-** | 7.48 | **-** | 17.79 | **-** |
| Std. Dev. | 3.69E-06 | **-** | 3.27 | **-** | 0.0433 | **-** | 0.0382 | **-** |
| Mean | 0 | **-** | 35.57 | **-** | 2.5 | **-** | 0.8782 | **-** |
| C.V. % | 9.35 | **-** | 9.2 | **-** | 1.73 | **-** | 4.35 | **-** |
| R² | 0.97 | **-** | 0.97 | **-** | 0.998 | **-** | 0.99 | **-** |
| Adjusted R² | 0.96 | **-** | 0.96 | **-** | 0.9952 | **-** | 0.99 | **-** |
| Predicted R² | 0.94 | **-** | 0.94 | **-** | NA⁽¹⁾ | **-** | 0.70 | **-** |
| Adeq Precision | 45.10 | **-** | 45.67 | **-** | 86.9935 | **-** | 109.99 | **-** |

**Table S 10 |** Regression coefficients of the mathematical models used to predict the microbial properties of OJ and DBLJ treated with CTS.

|  | DBLJ | | | | OJ | | | |
| --- | --- | --- | --- | --- | --- | --- | --- | --- |
|  | PEM activity | RA | TBC | D-Value | PEM activity | RA | TBC | D-Value |
| intercept | 0.0014 | 847.13 | 902.31 | 1018.66 | 0.00093 | 834.30 | 1010.77 | 596.47 |
| A | -0.00077 | -463.81 | -1071.49 | -1136.99 | -0.000509 | -457.28 | -1195.83 | -653.99 |
| B | 0.0022 | 1324.33 | -73461.83 | -1660.06 | 0.001365 | 1237.84 | -214.08 | 19617.60 |
| C | -7.7E-07 | -0.56 | -41.38 | -27.29 | -0.00000062 | -0.55 | -33.06 | -19.74 |
| AB | - | - | 88257.57 | -4466.69 | - | - | 98.98 | -29517.46 |
| AC | - | - | 49.14 | 31.59 | - | - | 39.63 | 23.02 |
| BC | - | - | -69.41 | 11.39 | - | - | -126.42 | -4.83 |
| A^2^ | - | - | 319.08 | 318.30 | - | - | 354.78 | 179.74 |
| B^2^ | - | - | 7352740 | 403508 | - | - | - | 475953 |
| C^2^ | - | - | 0.000067 | 0.000208 | - | - | - | -0.000419 |
| ABC | - | - | 41.49 | -4.96 | - | - | 75.49 | 4.21 |
| A^2^B | - | - | -26501.01 | 2962.74 | - | - | - | 10340.66 |
| A^2^C | - | - | -14.59 | -9.16 | - | - | -11.87908 | -6.71137 |
| AB^2^ | - | - | -8677050 | -223068 | - | - | 118150 | -265398 |
| A^2^B^2^ | - | - | 2559440 | - | - | - | -69537.14 | - |

**Table S11 |** Effect of optimization conditions on the number of microorganisms compared to TP in the fresh sample.

| Microbes  (log cfu/ml) | OJ | | | DBLJ | | |
| --- | --- | --- | --- | --- | --- | --- |
|  | **CT-OJ** | **TP** | **fresh** | **CT-DBLJ** | **TP** | **Fresh** |
| PS | ND^b^ | ND^b^ | 2.38 ± 0.14^a^ | ND^b^ | ND^b^ | 2.62 ± 0.07^a^ |
| CB | ND^b^ | ND^b^ | 2.07 ± 0.12^a^ | ND^b^ | ND^b^ | 2.21 ± 0.15^a^ |
| Y & M | ND^b^ | ND^b^ | 2.53 ± 0.09^a^ | ND^b^ | ND^b^ | 2.76± 0.11^a^ |

Ps: Psychrotrophes, CB: Coliform bacteria, Y&M: yeasts and molds , ND: Not detected ( < 1 CFU/mL).
